# Mild hyperlipidemia in mice aggravates platelet responsiveness in thrombus formation and exploration of platelet proteome and lipidome

**DOI:** 10.1038/s41598-020-78522-9

**Published:** 2020-12-08

**Authors:** Johanna P. van Geffen, Frauke Swieringa, Kim van Kuijk, Bibian M. E. Tullemans, Fiorella A. Solari, Bing Peng, Kenneth J. Clemetson, Richard W. Farndale, Ludwig J. Dubois, Albert Sickmann, René P. Zahedi, Robert Ahrends, Erik A. L. Biessen, Judith C. Sluimer, Johan W. M. Heemskerk, Marijke J. E. Kuijpers

**Affiliations:** 1 Department of Biochemistry, Cardiovascular Research Institute Maastricht (CARIM), Maastricht University, P.O. Box 616, 6200 MD Maastricht, The Netherlands; 2 Leibniz Institut für Analytische Wissenschaften – ISAS– e.V, Dortmund, Germany; 3 Department of Pathology, CARIM, Maastricht University, Maastricht, The Netherlands; 4 Department of Haematology, Inselspital, University of Bern, Bern, Switzerland; 5 Department of Biochemistry, University of Cambridge, Cambridge, UK; 6 The M-Lab, Department of Precision Medicine, School for Oncology and Developmental Biology (GROW), Maastricht University, Maastricht, The Netherlands; 7 Segal Cancer Proteomics Centre, Jewish General Hospital, McGill University, Montreal, Canada; 8 Department of Analytical Chemistry, Faculty of Chemistry, University of Vienna, Wien, Austria; 9 Institute for Molecular Cardiovascular Research, RWTH Aachen University, Aachen, Germany; 10 BHF Centre for Cardiovascular Science, University of Edinburgh, Edinburgh, UK

**Keywords:** Cardiovascular diseases, Platelets

## Abstract

Hyperlipidemia is a well-established risk factor for cardiovascular diseases. Millions of people worldwide display mildly elevated levels of plasma lipids and cholesterol linked to diet and life-style. While the prothrombotic risk of severe hyperlipidemia has been established, the effects of moderate hyperlipidemia are less clear. Here, we studied platelet activation and arterial thrombus formation in *Apoe*^−/−^ and *Ldlr*^−/−^ mice fed a normal chow diet, resulting in mildly increased plasma cholesterol. In blood from both knockout mice, collagen-dependent thrombus and fibrin formation under flow were enhanced. These effects did not increase in severe hyperlipidemic blood from aged mice and upon feeding a high-fat diet (*Apoe*^−/−^ mice). Bone marrow from wild-type or *Ldlr*^−/−^ mice was transplanted into irradiated *Ldlr*^−/−^ recipients. Markedly, thrombus formation was enhanced in blood from chimeric mice, suggesting that the hyperlipidemic environment altered the wild-type platelets, rather than the genetic modification. The platelet proteome revealed high similarity between the three genotypes, without clear indication for a common protein-based gain-of-function. The platelet lipidome revealed an altered lipid profile in mildly hyperlipidemic mice. In conclusion, in *Apoe*^−/−^ and *Ldlr*^−/−^ mice, modest elevation in plasma and platelet cholesterol increased platelet responsiveness in thrombus formation and ensuing fibrin formation, resulting in a prothrombotic phenotype.

## Introduction

Hyperlipidemia initiates, drives and aggravates atherosclerotic plaque formation, as an underlying cause for cardiovascular diseases^1,2^. In human adults, low plasma levels of cholesterol and triglycerides are highly desirable^3,4^. In 2015 however, 95 million adult Americans were presenting with elevated levels (> 200 mg/dl) of total cholesterol^5^. Similarly, in Europe the high prevalence of high plasma cholesterol is a serious health problem^6^. The relationship between hyperlipidemia, atherosclerosis and thrombosis has been extensively investigated^7,8^. Already in 1975, it was suggested that hypercholesterolemia in monkeys predisposes to increased platelet aggregation^9^. However, the majority of the studies investigating the effects of hyperlipidemia on atherothrombosis were performed in genetically modified mice, fed a high fat diet^10^. Depending on the genotype—*Apoe*^−/−^ or *Ldlr*^−/−^—feeding with a high fat diet causes plasma cholesterol and triglycerides levels increase to levels at least 5–tenfold higher than observed in hyperlipidemic individuals (> > 240 mg/dL)^11,12^. These levels are well known to induce plaque formation with ensuing atherothrombotic effects. However, much less is known about the effects of less aggressive forms of hyperlipidemia, that align with those seen in humans (mildly increased cholesterol levels of 200–239 mg/dL), on the thrombosis propensity and platelet activation.

Next to absolute cholesterol levels, also lipoprotein profiles of mice are distinct from man, in whom cholesterol is transported by low-density lipoproteins^12^. The *Apoe*^−/−^ mice carry plasma cholesterol in majority as very low-density lipoproteins and chylomicrons. The lipoprotein profile of *Ldlr*^−/−^ mice resembles more that of human hypercholesterolemia, with cholesterol preferentially transported by low-density lipoproteins^12,13^. Both knockouts develop progressive atherosclerosis of the aortic arch and carotid arteries, although the precise mechanism differs between the genotypes^10,14–17^, in that atherosclerotic lesion formation upon LDLR deficiency more relies on the dietary fat content^11^.

In the present study, we aimed to investigate how a moderate elevation of the plasma cholesterol influences platelet activation and thrombus formation. For this purpose, we adapted two multiparameter ex vivo methods of shear-dependent whole blood thrombus formation for murine blood, to study *(1)* platelet activation and aggregation at a collagen surface and *(2)* the kinetics of collagen/tissue factor (TF)-induced thrombus and fibrin clot formation. The mice were fed a normal chow diet, in order to provoke mildly increased plasma cholesterol levels in *Apoe*^−/−^ and *Ldlr*^−/−^ mice. We detected a platelet-dependent increase in thrombotic tendency using blood from both *Apoe*^−/−^ and *Ldlr*^−/−^ mice, compared with normocholesterolemic wild-type mice. These effects were not further aggravated in aged mice, nor in high-fat diet fed *Apoe*^−/−^ mice with 50% higher plasma cholesterol levels. Exploration of the platelet proteome and lipidome revealed additional insight into the platelet factors contributing to this prothrombotic phenotype of platelets.

## Results

### Multi-parameter assessment of platelet prothrombotic phenotype under flow in *Ldlr*^−/−^ and *Apoe*^−/−^ mice

**Figure 1:**
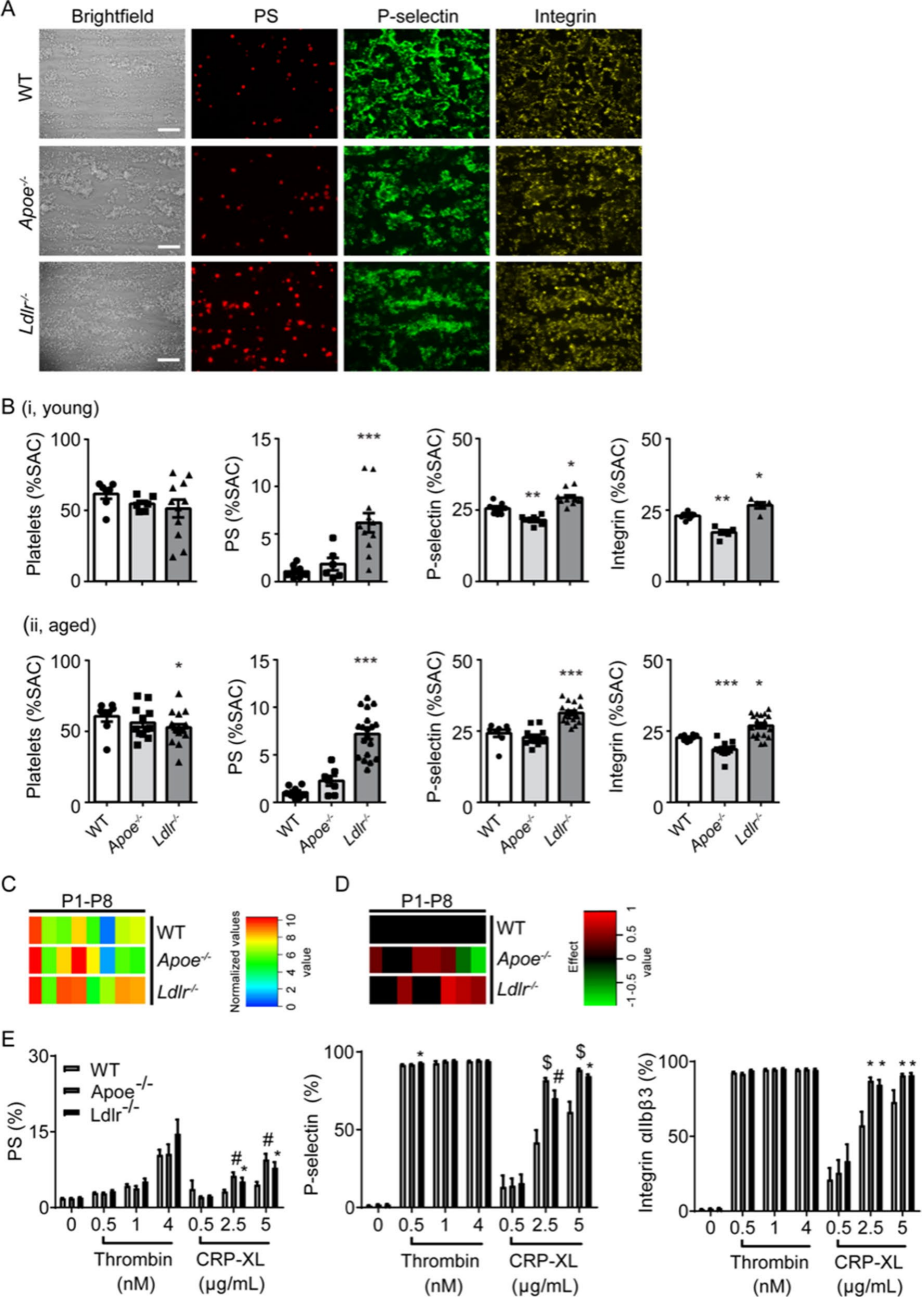
Multiparameter assessment of platelet activation and aggregation under arterial flow of *Apoe*^−/−^ and *Ldlr*^−/−^ mouse blood in the absence of coagulation. Blood was used from young (9–12 weeks) and aged (37–42 weeks) *Apoe*^−/−^, *Ldlr*^−/−^ and corresponding wild-type mice, held on normal chow diet. PPACK-anticoagulated blood was perfused over collagen type I at wall shear rate of 1000 s^−1^. Brightfield images were captured, after which the deposited platelets were stained for integrin α_IIb_β_3_ activation, P-selectin expression and PS exposure in different colors (see “Methods”). (**A**) Representative images from young mice are shown for each color per genotype. Bars indicate 20 μm. (**B**) Quantification (% SAC) of brightfield images of platelet deposition and fluorescence images of platelet activation parameters: phosphatidylserine (PS) exposure, P-selectin expression and integrin α_IIb_β_3_ activation of (i) young mice and (ii) aged mice. Mean ± SEM (*n* = 6–15 animals/group). *P < 0.05, **P < 0.01 and ***P < 0.001 vs. wild-type (Mann–Whitney *U* test). (**C**) Platelet activation parameters were obtained from brightfield and fluorescence images after 3.5 min: *P1*, morphological score; *P2*, platelet surface area coverage (% SAC); *P3*, aggregate contraction score; *P4*, aggregate multilayer score; *P5*, aggregate multilayer coverage (% SAC); *P6*, PS exposure (% SAC); *P7*, P-selectin expression (% SAC); *P8*, integrin α_IIb_β_3_ activation (% SAC). Values per parameter were linearly scaled to 0–10 and a scaled heatmap with integration of age groups was generated. (**D**) Scaled heatmap of effect sizes per genotype. Effect size per parameter was calculated from the pooled standard deviation, Cohen’s *d* and regression coefficient *r.* Analysis based on relevant changes restricted to differences with *P* < 0.05 (*t* test, 2-sided, equal variance). Means (*n* = 15–20 animals/group). (**E**) Isolated platelets were activated by thrombin (0.5–4 nM) or cross-linked collagen-related peptide (CRP-XL, 0.5–5 μg/ml); exposure of phosphatidylserine (PS), P-selectin and activation of integrin α_IIb_β_3_ were determined by flow cytometry using AF647-labeled annexin A5, FITC-labeled anti-CD62P mAb, and PE-labeled JON/A mAb, respectively. Means ± SEM (*n* = 8–12), *P < 0.05, ^#^*P* < 0.01, ^$^*P* < 0.001 compared to WT (Kruskall–Wallis test).

To investigate how platelet functions are modified under mildly hyperlipidemic conditions in both *Apoe*^−/−^ or *Ldlr*^−/−^ mice^16,18^, we adapted a previously validated multiparameter test for the simultaneous assessment of platelet adhesion and activation in human blood^19^. Accordingly, using microfluidics, recalcified whole blood from wild-type, *Apoe*^−/−^ and *Ldlr*^−/−^ mice of 9–12 weeks old, held on normal chow diet, was perfused over collagen micro-spots. Analysis of the platelet deposition on collagen did not show differences in adhesion between the genotypes (Fig. 1A,Bi). However, the thrombi that were generated with blood from *Apoe*^−/−^ or *Ldlr*^−/−^ mice appeared denser in structure and more layered compared to the wild-type mice (Fig. 1A). The difference was also seen as an increased thrombus contraction and aggregate multilayer score (Fig. 1C, see P3 and P4). In addition, specific platelet activation markers (PS exposure, P-selectin expression and integrin α_IIb_β_3_ activation) were increased for the *Ldlr*^−/−^ mice, compared to wild-types (Fig. 1B). However, for *Apoe*^−/−^ mice, PS exposure remained unchanged, and other activation markers were slightly reduced (Fig. 1Bi).

To investigate whether the thrombus-forming process was influenced by the (lipid) composition of the blood, blood samples were analyzed from both young and aged wild-type, *Apoe*^−/−^ and *Ldlr*^−/−^ mice. Overall, similar genotype-induced differences were observed for platelet adhesion and activation parameters for the aged animals as compared to the young mice (Fig. 1Bii, suppl. Figure 1A). Hematological parameters indicated a 35% lower platelet count in blood from all *Ldlr*^−/−^ mice, but not *Apoe*^−/−^ mice, compared to wild-types (Suppl. Table 1). Across mouse strains, platelet count increased by 25–45% upon ageing, in agreement with published findings^20^. Red and white blood cell counts were similar between genotypes and age groups. It has been reported that *Apoe*^−/−^ mice develop leukocytosis, especially when receiving a high-fat diet^11^. In our study with mice on normal chow diet, numbers of leukocytes were similar in all genotypes (Suppl. Table 1). However, in *Apoe*^−/−^ mice that were held on high-fat diet (Suppl. Figure 2), we noted a significant increase in leukocyte counts from 3.8 ± 0.8 × 10^3^/µl to 6.1 ± 2.4 × 10^3^/µl (mean ± SD, *n* = 10–12, *P* < 0.01). In comparison to wild-type mice, plasma cholesterol levels were more elevated in aged *Apoe*^−/−^ mice (sixfold) than in aged *Ldlr*^−/−^ mice (threefold) (Suppl. Table 1).

**Figure 2:**
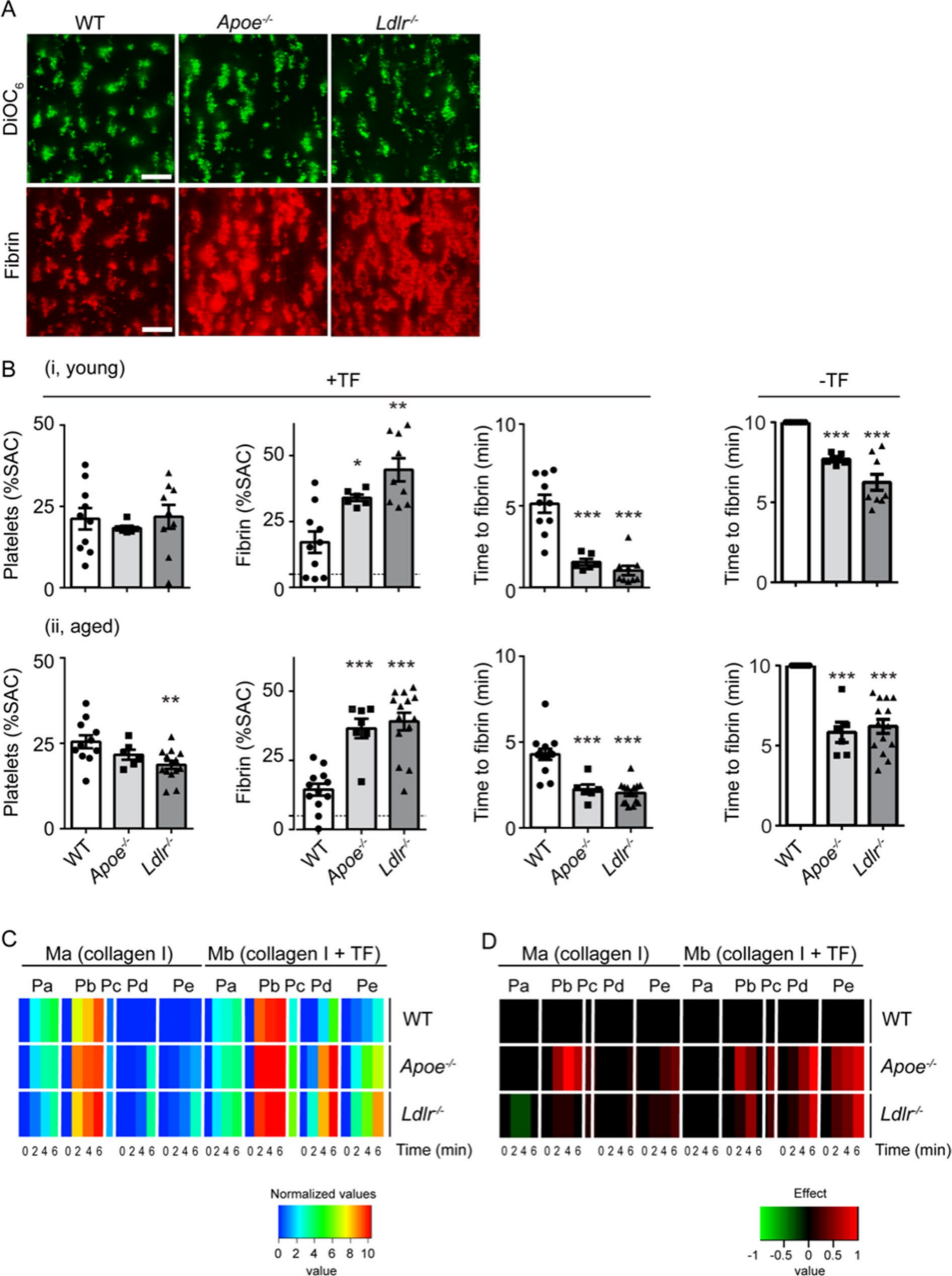
Deficiency in ApoE or LDLR enhances formation of platelet–fibrin thrombi in the presence of coagulation at arterial flow rate. Blood was obtained from young (9–12 weeks) and aged (37–42 weeks) *Apoe*^−/−^, *Ldlr*^−/−^ and corresponding wild-type mice held on normal chow diet. Citrated blood samples were supplemented with DiOC_6_ (labeling platelets) plus AF647 fibrinogen, and co-perfused with CaCl_2_/MgCl_2_ over two microspots consisting of collagen (microspot *Ma*) and collagen/TF (microspot *Mb*) at a wall shear rate of 1000 s^−1^. Confocal two-color fluorescence images were captured in real time at 45 s time intervals. (**A**) Representative images of deposited platelets (DiOC_6_, green) and fibrin (AF647, red) from young mice at spot *Mb* after 4 min; bars = 20 µm. (**B**) Quantification of surface area coverage (% SAC) of platelet deposition, and fibrin-covered area at spot *Mb* after 4 min; horizontal dotted line indicates labeling threshold for fibrin formation, as well as quantification of time to first fibrin formation of (i) young mice and (ii) aged mice. Left part: whole blood was perfused over collagen co-coated with tissue factor (+ TF), right part: Time to first fibrin formation in whole blood perfused over collagen without tissue factor (− TF). (**C**) Thrombus parameters were obtained from brightfield and fluorescence images at time points 0, 2, 4 and 6 min: *Pa*, DiOC_6_ platelet deposition (% SAC); *Pb*, DiOC_6_ platelet thrombus score (0–5); *Pc*, time to first fibrin formation (− log min); *Pd*, AF647 fibrin score; *Pe*, AF647 fibrin-covered area (% SAC). Values per parameter were linearly scaled to 0–10. Presented is a scaled heatmap of the integrated age groups (9–12 weeks and 37–42 weeks, see suppl. Figure 3). (**D**) Scaled heatmap of changes per genotype, filtered for moderate effect size. Effect size per parameter was calculated from pooled standard deviation, Cohen’s *d* and regression coefficient *r.* Analysis based on relevant changes restricted to differences with *P* < 0.05 (*t* test, 2-sided, equal variance). Means (*n* = 13–23 animals/group).

A heatmap was generated for a more systematic analysis of the eight thrombus parameters, which were averaged and scaled per parameter (Suppl. Figure 1B). Statistical analysis indicated that only 1 out of 8 parameters differed between the age groups of *Ldlr*^−/−^ mice, and that none of the parameters differed between the young and old *Apoe*^−/−^ mice. This allowed us to pool the values from either age group per genotype (Fig. 1C). The effect-size differential heatmap of Fig. 1D, obtained by filtering differences from wild-types against significance (*P* < 0.05), then pointed to a clear prothrombotic phenotype of the blood from both knockout mice, albeit with a different pattern of increased parameters. The *Apoe*^−/−^ mice showed predominantly increased platelet aggregation parameters (morphological score, multilayer score and platelet aggregate coverage) (Fig. 1D); while the *Ldlr*^−/−^ mice showed elevated platelet aggregation as well as activation markers (PS exposure, P-selectin expression and integrin activation).

Flow cytometry analysis with isolated platelets confirmed that platelet activation was increased upon GPVI stimulation, when comparing the knockouts with wild-types (Fig. 1E). In an additional study, *Apoe*^−/−^ mice were fed a high fat diet, increasing plasma cholesterol by 50% (Suppl. Figure 2A). Markedly, this diet did not further increase the prothrombotic phenotype upon ApoE deficiency, as detected by either microfluidics tests or flow cytometry of platelet activation with a panel of agonists (Suppl. Figure 2B,C). Given that flow perfusion over collagen is a relevant surface for identifying changes in murine arterial thrombosis^21,22^, we concluded that the platelets from both *Apoe*^−/−^ and *Ldlr*^−/−^ mice carry a prothrombotic phenotype, with *Apoe*^−/−^ platelets showing predominantly an increased aggregation tendency and *Ldlr*^−/−^ platelets showing an increased secretion.

### Enhanced platelet-dependent fibrin and thrombus formation under flow in *Ldlr*^−/−^ and *Apoe*^−/−^ mice

To investigate whether the increased platelet responses of *Apoe*^−/−^ and *Ldlr*^−/−^ mice extended to coagulation stimulation, *i.e.* fibrin formation, we adapted a method to assess the kinetics of this process using perfused human blood^23^. Therefore, recalcified mouse blood was labeled with DiOC_6_ and AF647-fibrinogen, and perfused through a flow chamber over two microspots consisting of collagen type I (upstream microspot *Ma*) and collagen type I + TF (downstream microspot *Mb*).

Whole blood flow under coagulating conditions resulted in similar platelet deposition for all three genotypes on collagen/TF (Fig. 2A,Bi), although a slight but significant decrease in platelet deposition was observed for aged *Ldlr*^−/−^ mice compared to the corresponding wild-types (Fig. 2Bii). Using blood from young or aged wild-type mice, the co-coating of TF significantly enhanced platelet deposition on collagen (Fig. 2B vs. Suppl. Figure 4B, *P* < 0.001). With blood from *Apoe*^−/−^ or *Ldlr*^−/−^ mice, platelet deposition in the absence of TF was enhanced after 4–6 min, when compared to wild-types (Suppl. Figure 4A, D), while TF did not further enhance this parameter (Fig. 2B). Accordingly, in contrast to wild-type blood, there was measurable fibrin formation in the absence of TF with blood from *Apoe*^−/−^ or *Ldlr*^−/−^ mice (Fig. 2B, Suppl. Figure 4C). At the TF-containing spot *Mb*, 2–3 times more fibrin was formed when using blood from *Apoe*^−/−^ or *Ldlr*^−/−^ mice than with wild-type blood (Fig. 2A,B). In agreement with this, the time to fibrin formation was shortened in blood from either knockout mouse (Fig. 2B). Of note, in the absence of TF, no fibrin was formed in wild-type mice within 10 min, while in all young and aged knockout mice fibrin was generated within 10 min (Fig. 2B). No differences were recorded between young or old mice, regardless of the genotype (Fig. 2, Suppl. Figures 3 and 4). Kinetic analysis of platelet deposition in the presence of TF did not reveal differences between genotypes or age groups, while fibrin deposition in time was markedly enhanced for both *Apoe*^−/−^ and *Ldlr*^−/−^ mice compared to wild-type mice (Suppl. Figure 3B). To determine the rate of fibrin formation, the slope of each curve was calculated over the steepest part (1.5–3.75 min). For wild-type blood, the maximum slope was 4.8%SAC/min. For *Apoe*^−/−^ blood, this slope increased to 8.5%SAC/min (*P* < 0.05 vs. wild-type). For *Ldlr*^−/−^ mice this value further elevated to 13.4%SAC/min (*P* < 0.0005 vs. wild-type).

**Figure 3:**
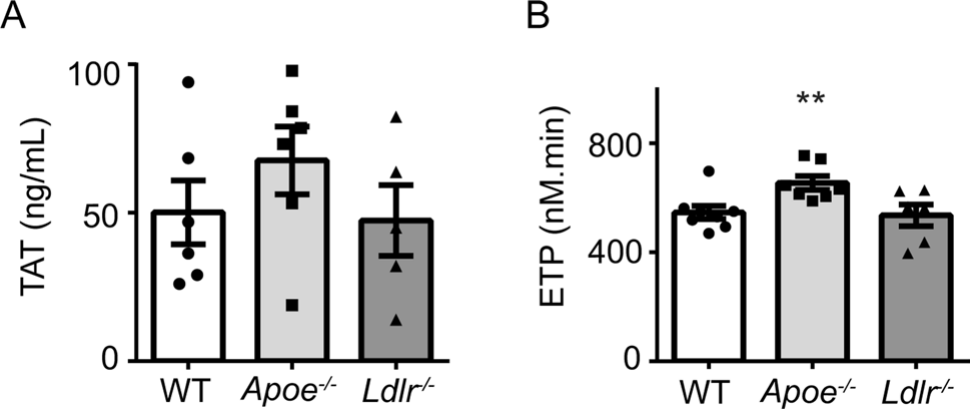
No relevant differences in coagulation parameters measured in plasma from *Apoe*^−/−^ and *Ldlr*^−/−^ mice. (**A**) Circulating thrombin-antithrombin (TAT) complexes measured in plasma from wild-type, *Apoe*^−/−^ and *Ldlr*^−/−^ mice on normal chow diet. Means ± SEM (*n* = 5–6 animals/group, Mann–Whitney *U* test). (**B**) Thrombin generation in plasma from *Apoe*^−/−^ and *Ldlr*^−/−^ mice. Calibrated automated thrombin generation was assessed at 1 pM tissue factor (f.c.) in platelet-free plasma from wild-type, *Apoe*^−/−^ and *Ldlr*^−/−^ mice. Standard curve parameters were recorded: shown is endogenous thrombin potential (ETP, area-under-the-curve) as means ± SEM (*n* = 6–8). **P < 0.01 vs. wild-type (Mann–Whitney U test). Lag time to initial thrombin, thrombin peak height, and time-to-thrombin peak were not significantly different between groups (not shown).

**Figure 4:**
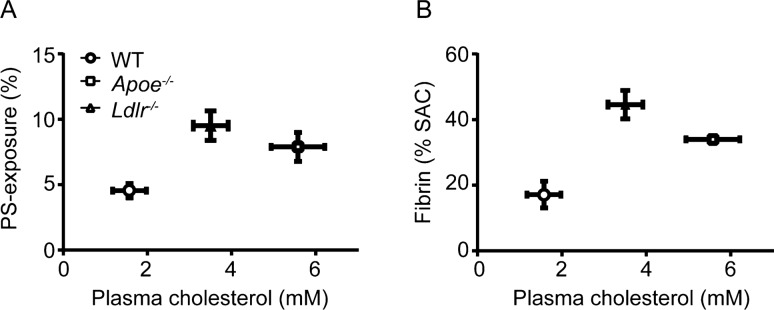
Plasma cholesterol level is associated with platelet phosphatidylserine exposure and platelet-induced fibrin formation. Plasma cholesterol level (Table 1) from wild-type, *Apoe*^−/−^ and *Ldlr*^−/−^ mice plotted against: (**A**) exposure of phosphatidylserine (PS) in washed platelets stimulated with 5 mg/ml CRP-XL (see Fig. 1E) and (**B**) surface area coverage (% SAC) of fibrin-covered area from whole blood perfusion over collagen/TF (Fig. 2B).

To allow a direct comparison of all differences in thrombus formation, a heatmap was generated using five outcome parameters (*Pa-e*), four of which were assessed at 0, 2, 4 and 6 min (Suppl. Figure 3C). Of the 34 obtained parameters of thrombus formation on two microspots, only 2–8 parameters showed significant differences between the mouse age groups of all genotypes (Suppl. Figure 3C). In agreement with previous data^24^, the various parameters per microspot were mostly correlated when examined per genotype (Fig. 2C). When comparing the knockout strains, gain-of-function values (represented as yellow–red coloring) were observed for both the *Apoe*^−/−^ and *Ldlr*^−/−^ mice, when compared to corresponding wild-types, especially for the fibrin parameters *Pc*, *Pd* and *Pe*.

A subtraction heatmap was created for better visualize of differences in terms of effect sizes per parameter (Fig. 2D). This revealed a similar prothrombotic phenotype in phosphatidylserine exposure and fibrin formation for both *Apoe*^−/−^ and *Ldlr*^−/−^ mice, especially in the presence of TF. This extended analysis hence suggested that the hyperlipidemic condition in either knockout strain led to an increase in platelet activation and ensuing fibrin formation under flow conditions.

To investigate if coagulation processes were altered, platelet-free plasma from the genotypes was analyzed for the presence of thrombin-anti-thrombin (TAT) complexes, as a sensitive marker of ongoing thrombin generation *in* vivo^25^. No significant differences between the three strains were seen (Fig. 3A). The TF-induced thrombin generation test in plasma showed a slightly increased endogenous thrombin potential for *Apoe*^−/−^, compared to wild-type or *Ldlr*^−/−^ plasma (Fig. 3B). This pointed to a weak enhancement of coagulant activity in only *Apoe*^−/−^ mice, which however cannot fully explain the increased platelet-dependent fibrin formation during perfusion of whole blood from both *Apoe*^−/−^ and *Ldlr*^−/−^ mice.

When relating per genotype the plasma cholesterol levels (Suppl. Table 1) with the extent of PS exposure in isolated platelets (Fig. 1E), there appeared to be a good correlation (Fig. 4A). Furthermore, this correlation extended to the amounts of fibrin formed (Fig. 4B). Although correlational, this analysis suggested a role of plasma cholesterol in the mildly hyperlipidemic mice in determining platelet-dependent coagulation.

### Hyperlipidemic environment promotes gain-of-platelet-function

**Figure 5:**
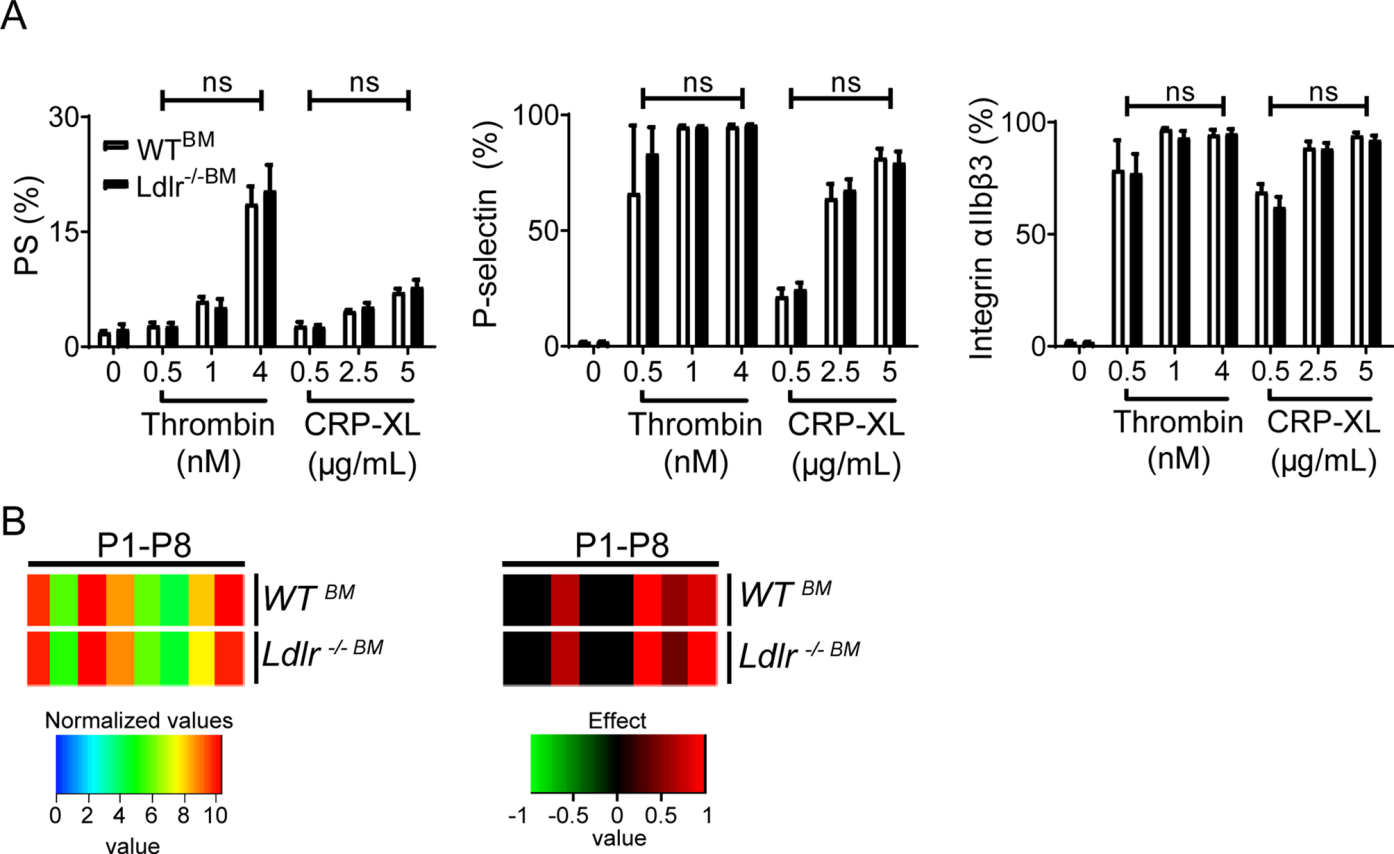
Similar activation of wild-type and Ldlr deficient platelets after transplantation of Ldlr-deficient mice. Bone marrow transplantations were performed using bone marrow from either wild-type *(WT*^*BM*^*)* or *Ldlr*^−/−^ mice *(Ldlr*^−/−*BM*^) injected into irradiated *Ldlr*^−/−^ recipients, held on normal chow diet. (**A**) Isolated platelets were activated by thrombin (0.5–4 nM) or cross-linked collagen-related peptide (CRP-XL, 0.5–5 μg/ml), and exposure of PS, P-selectin and activation of integrin α_IIb_β_3_ were determined by flow cytometry using AF647-labeled annexin A5, FITC-labeled anti-CD62P mAb, or PE-labeled JON/A mAb, respectively. Means ± SEM (*n* = 4–8 animals/group). (**B**) PPACK-anticoagulated blood was perfused over collagen type I at wall shear rate of 1000 s^−1^. Brightfield images were captured, after which the deposited platelets were stained for integrin α_IIb_β_3_ activation, P-selectin expression and PS exposure in different colors. Platelet activation parameters were obtained, as described for Fig. 1. Left: Scaled heatmap with integration of age groups. Right: Scaled heatmap of effect sizes per genotype. Effect size per parameter was calculated from the pooled standard deviation, Cohen’s *d* and regression coefficient *r.*

To provide more direct evidence for the influence of plasma cholesterol on platelets in these mice, we then performed a transplantation study. Bone marrow obtained from either wild-type or *Ldlr*^−/−^ donor mice was injected into whole-body irradiated *Ldlr*^−/−^ recipient mice, and the mouse blood samples were analyzed at 6 weeks after transplantation. Repopulation of the bone marrow with transplanted cells was confirmed in DNA extracted from white blood cells (92 ± 2% LDLR chimerism). In addition, plasma cholesterol levels were checked, and appeared to be equally high in both groups (Suppl. Table 2). Flow cytometry analysis of the blood samples demonstrated a similar, high platelet activation tendency of the *WT*^*BM*^ and *Ldlr*^−/−*BM*^ platelets (Fig. 5A). Flow perfusion experiments on collagen I indicated a similar gain-of-platelet-function for both transplantation arms, *i.e.* similar to the blood from full *Ldlr*^−/−^mice, when compared to wild-type blood (Fig. 5B). This provided a clear indication that the hyperlipidemic environment in the recipient *Ldlr*^−/−^ mice provoked a gain-of-platelet function of wild-type platelets, to reach the prothrombic activity of *Ldlr*^−/−^ platelets.

### Minor common changes in proteome of platelets from *Ldlr*^−/−^ and *Apoe*^−/−^ mice

**Figure 6:**
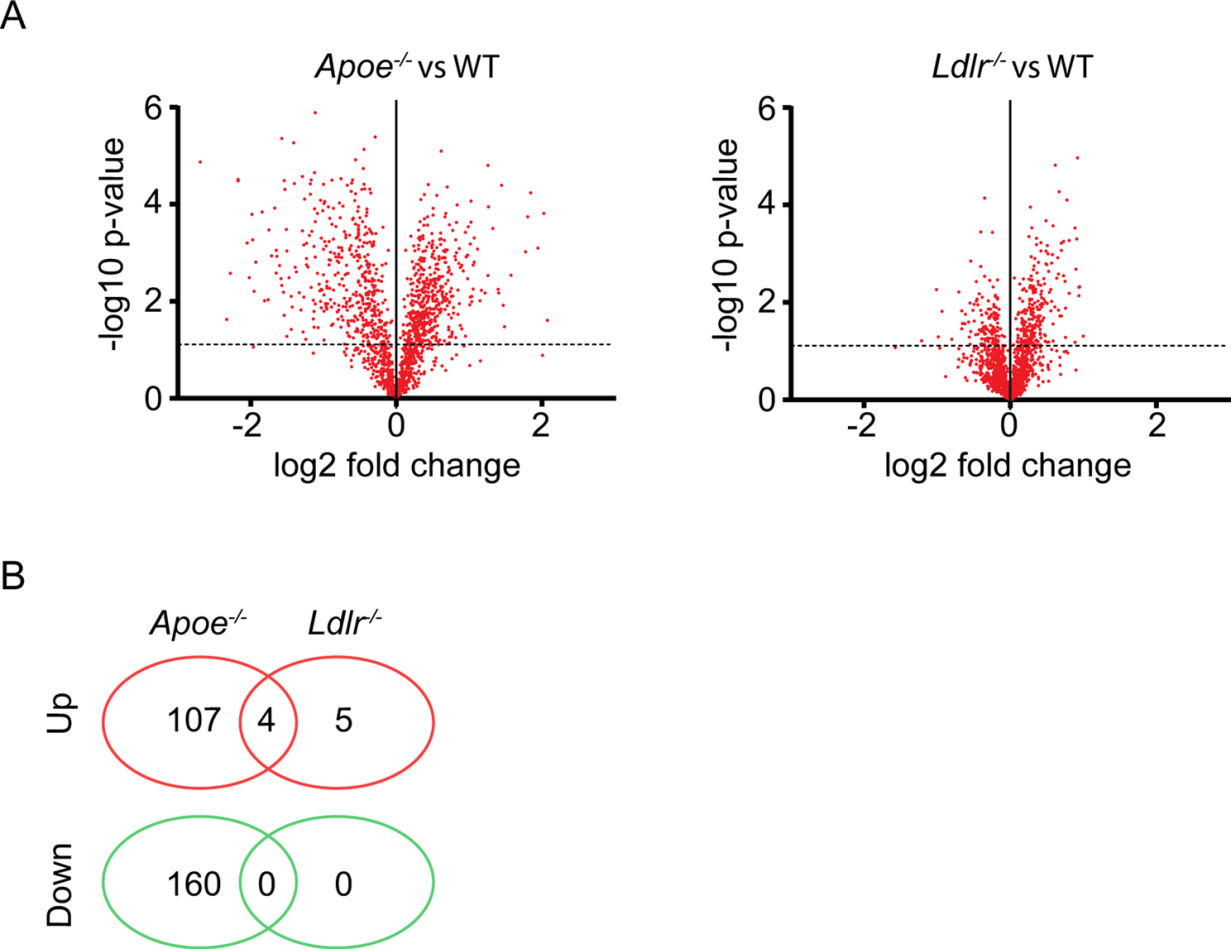
Deficiency in ApoE or LDLR results in limited changes in platelet proteome. Highly purified platelets from wild-type, *Apoe*^−/−^ and *Ldlr*^−/−^ mice were used for quantitative proteome analysis after shot-gun proteolysis. Ratios of 1,533 unique proteins were quantified by label-free proteomics analysis. (**A**) Median peptide ratios per protein were log2 transformed and plotted as a function of − log10 *P* values. Depicted are volcano plots comparing platelets from (**A**) *Apoe*^−/−^ with corresponding wild-type mice, and (**B**) *Ldlr*^−/−^ with wild-type mice. Dotted line indicates *P* = 0.05 (*t* test). (**C**) Venn diagram of number of proteins with a > 1.5-fold down- (green) or upregulation (red) in platelets from *Apoe*^−/−^ and *Ldlr*^−/−^ mice, as compared to wild-types.

Using quantitative proteomics analysis, we explored the protein composition of *Apoe*^−/−^ and *Ldlr*^−/−^ platelets in comparison to wild-type platelets. From platelet samples of all genotypes, ratio information was obtained regarding 1,533 unique proteins. Volcano plots indicated differences in ratios for multiple identified proteins between wild-type and *Apoe*^−/−^ or *Ldlr*^−/−^ platelets (Fig. 6A). Statistics after correction for multiple testing pointed to 655 and 21 significant protein changes in the *Apoe*^−/−^ and *Ldlr*^−/−^ platelets, respectively. Of these proteins, only a few showed relevant changes, *i.e.* > 1.5-fold as previously defined^26,27^. For *Apoe*^−/−^ platelets this concerned 277 proteins (18%), and for *Ldlr*^−/−^ platelet 9 proteins (0.6%) (Fig. 6B and Suppl. Table 3). Remarkably, only 4 proteins were jointly upregulated and none were jointly downregulated in both knockout strains (Suppl. Table 3C).

Regarding cellular functions of the altered proteins^28,29^, these were confined to mostly structural proteins (Table 1). For *Apoe*^−/−^ mice downregulation was observed in several mitochondrial and metabolic proteins, while both up- and downregulation was seen of in particular reticular, membrane and signaling-related proteins. Specifically, in *Ldlr*^−/−^ platelets, secretory proteins were upregulated compared to wild-type platelets. However, close inspection of the (moderately) altered proteins did not reveal candidates for a common increase in platelet activation tendency for the two hyperlipidemic mouse strains.

**Table 1:**
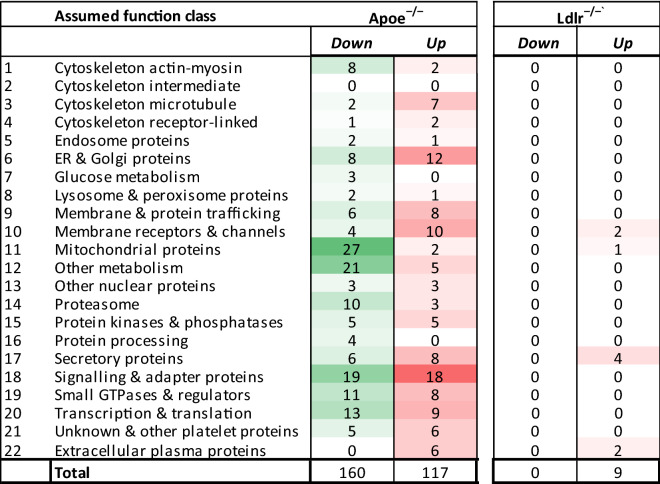
Overview of differences in quantitative proteome of ApoE- and LDLR-deficient platelets as compared to wild-type platelets. Highly purified platelets from wild-type, *Apoe*^−/−^ and *Ldlr*^−/−^ mice were subjected to label-free proteomics analysis. Ratios of 1,533 unique proteins were quantified by mass spectrometry (see “Methods”). Unique proteins of the quantitative proteome were assigned to 22 assumed function classes. Green represents > 1.5 fold downregulated and red > 1.5-fold upregulated proteins. *N* = 3–4 animals/group.

### Changes in platelet lipid profiles of ApoE and LDLR deficient mice

**Figure 7:**
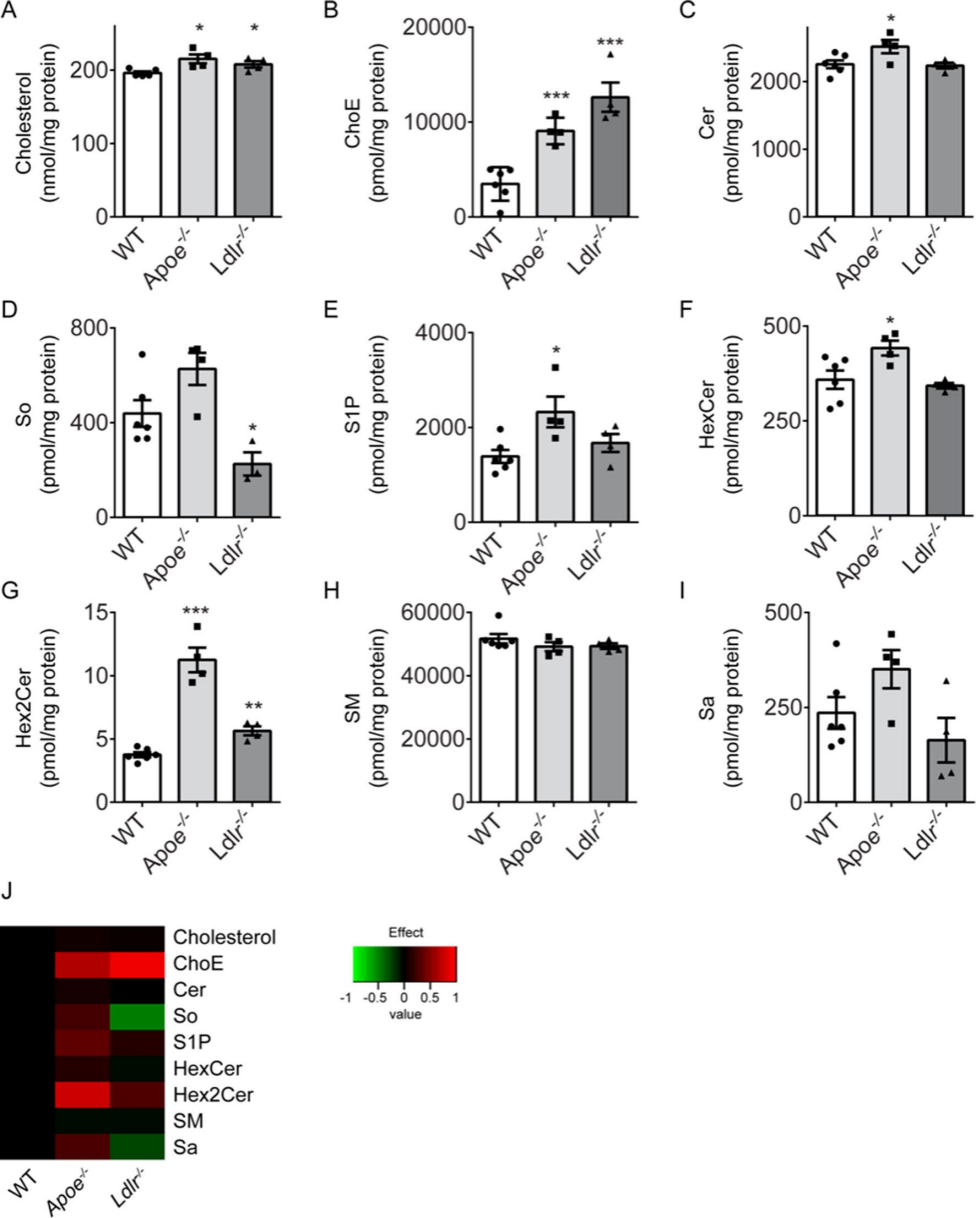
Alterations in lipid profile in platelets from *Apoe*^−/−^ and *Ldlr*^−/−^ mice. Highly purified platelets from wild-type, *Apoe*^−/−^ and *Ldlr*^−/−^ mice were used for quantitative lipidomic analysis. (**A**) Cholesterol and (**B**) cholesteryl esters (ChoE); (**C**) ceramides (Cer); (**D**) sphingosine (So); **E**) sphingosine-1-phosphate (S1P); **F**) glycosylceramide or galactosylceramide (HexCer); (**G**) lactosylceramide (Hex2Cer); (**H**) Sphingomyelin (SM); (**I**) sphinganine (Sa). Means ± SEM (*n* = 4–6 animals/group), *P < 0.05, **P < 0.01, ***P < 0.001 (Mann–Whitney U test). (**J**) Cluster analysis of quantitative lipidome analysis. Significantly altered lipid classes of *Apoe*^−/−^ or *Ldlr*^−/−^ platelets compared to wild-type platelets (log2 transformed) are indicated in red (increased) or in green (decreased).

Given the common, moderate hyperlipidemic phenotype of both mouse strains (Suppl. Table 1), we also assessed the lipidomic profiles of *Apoe*^−/−^, *Ldlr*^−/−^ and wild-type platelets. Quantitative analysis of the lipid classes indicated that especially free cholesterol and cholesteryl esters were elevated in platelets from *Apoe*^−/−^ as well as *Ldlr*^−/−^ mice (Fig. 7A,B). Additional differences in comparison to wild-type platelets were observed for specific sphingolipids. The sphingolipid precursor ceramide was significantly increased in *Apoe*^−/−^ platelets (Fig. 7C). Ceramide incorporates into sphingosine and sphingosine-1-phosphate in the endoplasmic reticulum^30^, and both were also increased in *Apoe*^−/−^ platelets (Fig. 7D,E). In contrast, the level of sphingosine was downregulated in *Ldlr*^−/−^ platelets (Fig. 7D). Ceramide is also transported to the Golgi system, where the glycosphingolipids isoforms HexCer and Hex2Cer are formed^30^. Both isoforms were also significantly increased in *Apoe*^−/−^ platelets, but less so in *Ldlr*^−/−^ platelets (Fig. 7F,G). No differences were observed for other sphingolipids (Fig. 7H,I). Cluster analysis of the quantitative lipidome analysis highlighted the changes in lipid classes between the three genotype groups (Fig. 7J).

## Discussion

In this paper, we investigated the prothrombotic propensity and underlying processes of two (moderately) hyperlipidemic mouse strains, *i.e. Apoe*^−/−^ and *Ldlr*^−/−^ mice, fed a normal chow diet. Multiparameter measurements of whole blood thrombus and fibrin formation on collagen indicated an exacerbation of the thrombotic process in either knockout strain, when compared to the corresponding wild-type mice. These altered platelet properties were not further aggravated in aged mice, nor by a high-fat diet (*Apoe*^−/−^), *i.e.* conditions that further elevate the plasma cholesterol levels by 50%. This suggested that a moderate elevation in plasma cholesterol is sufficient to enhance platelet activation and ensuing fibrin formation. Thrombin generation curves and TAT complexes in plasma did not show differences between genotypes, except for a moderate increase in thrombin potential in *Apoe*^−/−^ mice, suggesting that the observed effects are primarily due to changes in the platelets themselves. Importantly, bone marrow transplantation of wild-type or *Ldlr*^−/−^ platelets into irradiated *Ldlr*^−/−^ recipient mice resulted in platelets with a similar gain-of-function in adhesion, activation and thrombus formation, indicating that the hyperlipidemic environment of *Ldlr*^−/−^ mice led to an overall increased activation tendency of the chimeric wild-type platelets. Exploration of the platelet proteome revealed overall high similarity between the three genotypes, although some proteins showed significantly increased or decreased expression, particularly in *Apoe*^−/−^ mice. Finally, platelets of the mildly hyperlipidemic mice had an altered lipid profile, which is likely to play a role in the observed prothrombotic phenotype.

In the absence of coagulation, both knockout mice demonstrated a prothrombotic phenotype in whole blood perfusion over collagen, but with a different pattern of increased parameters. In comparison to wild-types, *Apoe*^−/−^ platelets showed predominantly an increased aggregation tendency and *Ldlr*^−/−^ platelets showed increased secretion. Multiparameter measurements of thrombus formation on collagen/TF in the presence of coagulation indicated a strikingly similar enhancement of the thrombotic process in both knockout mice. This agrees with reports that both mouse strains display an aggravated arterial thrombosis tendency *in* vivo^16,18^. Remarkably, the enhancement was also seen on collagen microspots in the absence of TF, which indicates that the prothrombotic phenotype (both coagulation and platelets) has already reached a plateau, and is not further enhanced by TF. This agrees with a platelet-dependent rather than a TF-triggered mechanism of the prothrombotic propensity. Indeed, collagen has been established as a key component of (ruptured) atherosclerotic plaques driving arterial thrombosis in vivo and *in* vitro^31,32^. In addition, the observed increases in platelet activation may stimulate platelet adhesion to other components of the inflamed vessel wall at atherosclerotic plaques^33^.

In the blood from aged *Ldlr*^−/−^ mice, we observed a minor 6% reduction in platelet deposition on collagen, which related to a 35% lower platelet count (unchanged platelet volume) in these mice, when compared to wild-types. This reduction in platelets was unexpected, since other studies suggest that platelet production is promoted by hypercholesterolemia (reviewed by Wang et al.^34^). Normal values of murine platelet counts in the literature vary between 450 to 1690 × 10^9^/L^35^. Given this, the count in all three genotypes was within the normal range. Furthermore, the moderate platelet reduction in *Ldlr*^−/−^ does not seem of physiological relevance, since in mouse an 80% reduction in count is needed to affect in vivo arterial thrombus formation^36^. No relevant differences in leukocyte count were observed, other than an increase in *Apoe*^−/−^ mice maintained on high-fat diet, which agrees with a previous report^11^. Together, our findings are in support of the concept of reactive thrombopoiesis, meaning that megakaryocytes adapt to a changing (lipid) environment, thereby affecting the formed platelets^37,38^.

Several studies have reported an enhanced arterial thrombosis tendency in vivo in *Apoe*^−/−^ and *Ldlr*^−/−^ mice^16,18,39–41^. As we recently demonstrated in a synthesis paper, analyzing over 1400 studies with genetically modified mice, gene deletion effects on thrombus formation in vitro appeared to correlate significantly with the gene deletion effects on arterial thrombosis *in* vivo^42^. Accordingly, the current findings of a prothrombotic propensity in vitro of (platelets in) *Apoe*^−/−^ and *Ldlr*^−/−^ blood are fully in line with the earlier detected enhanced thrombosis in these mouse strains. Yet, in translational terms, a limitation of the present study is that *Apoe*^−/−^ and *Ldlr*^−/−^ mice are still genetic in nature and have a lipid profile that differs from the human situation^12^. Although murine platelets differ from human platelets with regard to size, and number, functionally they are very similar^35,43^. Furthermore, the interplay of platelet activation and coagulation appears to be remarkably similar between mouse and man^35,42,44^. Feeding of wild-type mice with a high-fat diet may be a more physiologically relevant model, although also wild-type mice have a dissimilar lipid profile as compared to humans. Few studies have shown that also in wild-type animals, feeding a high-fat diet results in a prothrombotic phenotype and altered coagulation profile^45–48^. Yet, a translation from mouse studies to the human situation should be treated with caution.

The more enhanced fibrin-thrombus formation of *Apoe*^−/−^ mice was accompanied by a limited enhancement of coagulant activity in plasma from these mice. Given the earlier detected alterations in coagulation factor levels in *APOE2* knock-in mice^49^, similar changes may be present in the current mouse strain, thus explaining the enhanced thrombin generation potential. Markedly, no alterations in plasmatic coagulation were observed in *Ldlr*^−/−^ mice^11^. However, viscosity and osmolality changes may also contribute to the plasma sensing of platelets in flow studies.

In mouse, hyperlipidemia has been linked to an increased platelet reactivity with proposed roles of oxidized LDL and of the receptor proteins, CD36 and P2Y_12_^16,50–52^. The present comparison of the platelet proteome of (mildly hyperlipidemic) *Apoe*^−/−^ and *Ldlr*^−/−^ mice provides an extension of the first published platelet proteome of wild-type (C57BL/6) platelets^53^. Using a > 1.5-fold change as criterium for altered protein abundance levels^26,27^, some proteins appeared to be up- or downregulated in the knockout platelets, with the changes in *Apoe*^−/−^  >  > *Ldlr*^−/−^. Changes were mostly confined to Golgi, membrane, signaling and secretory proteins. Regarding the latter class, abundance levels of thrombospondin-2 and complement factors C3 and C4 were upregulated in platelets both knockout mice, while also coagulation factor XIII, reticulon-4, and amyloid beta protein were significantly changed in the *Apoe*^−/−^ mice. By themselves, these proteins have been shown to influence platelet function as shown in several functional assays, including those used in the present study (Suppl. Table 4 and references therein). How these proteins in combination alter platelet function is unclear. Of special interest are the complement factors, as these are relevant in thrombo-inflammation^54^, as well as for platelet properties and shedding of microparticles (Suppl. Table 4 and references therein). A recent study showed that C3^−/−^ mice displayed a prolonged bleeding time, and reduced thrombus formation, fibrin and platelet deposition in the ligated inferior vena cava, and diminished platelet activation in vitro^55^. Furthermore, the binding of C3 to platelets promotes the formation of platelet-leukocyte aggregates^54^.

A major outcome of the platelet lipidome profiling was an increase in cholesterol species in both knock-out mice. In translational terms, this matches with the high cholesterol levels in platelets from patients with hypercholesterolemia^56^. Especially this finding is compatible with a prothrombotic phenotype, as this suggests alterations in membrane fluidity and an increase in membrane lipid rafts in the *Apoe*^−/−^ and *Ldlr*^−/−^ platelets^34^. Platelet signaling is indeed considered to be dependent on membrane raft formation^57,58^. Another difference in *Apoe*^−/−^ and *Ldlr*^−/−^ platelets compared to wild-types were (distinct) changes in levels of (glyco)sphingolipids. While an altered sphingolipid metabolism can modulate platelet activation^59^, precisely how is still unclear. Options in the current context are effects of sphingolipid species on lipid sorting in the Golgi apparatus, or effects on lipid degradation in the lysosomes.

Hyperlipidemia is associated with shortened platelet survival and increased turnover in man^34^ and mouse^37^. However, our bone marrow transplantation experiment, in combination with only minor changes in the total platelet proteome, suggests that altered platelet protein function, not platelet count, is a major factor in the prothrombotic effects of hyperlipidemia. These changes in protein activity can be caused by post-translational modifications (PTMs), which regulate protein activity and are also effective in platelets. These PTMs include phosphorylation, glycosylation, palmitoylation, ubiquitination and oxidative stress, all of which have been described to alter platelet proteins during hemostasis as well as in several diseases including hyperlipidemia^60^. Although both hyperlipidemic mouse strains show a prothrombotic phenotype, we also observed differences between the *Apoe*^−/−^ and *Ldlr*^−/−^ platelets in several measurements, as indicated above. One explanation for this may be the different lipoprotein profiles of *Apoe*^−/−^ and *Ldlr*^−/−^ mice, being mainly VLDL and chylomicrons particles or LDL, respectively^12^. The effects of (oxidized) LDL on platelet function has been well established^61^, and also some studies have shown that VLDL induces platelet aggregation^62,63^. As different classes of lipoproteins have been shown to differently affect (human) platelet activation, the dissimilar balance in lipoprotein classes between both knockout strains may also explain the observed subtle differences in platelet activation. Furthermore, we postulate that different PTMs may be induced by LDL and VLDL.

Taken together, we conclude that the prothrombotic phenotype of mouse platelets in a mildly hyperlipidemic environment is triggered by the lipid surroundings, resulting in moderate changes in lipid abundance levels. The positive priming of platelets in hyperlipidemia increases their responsiveness and enhances platelet-dependent coagulation and fibrin clot formation under flow. In a translational perspective, it will be important to extend the study to humans with moderately elevated cholesterol levels.

## Methods

### Animals

All animal work was approved by the Maastricht University Animal Ethics Committee and the animal experiments were performed conform the guidelines from Directive 2010/63/EU of the European Parliament on the protection of animals used for scientific purposes. Wild-type C57BL/6J, *Apoe*^−/−^ and *Ldlr*^−/−^ mice (Charles River, Sulzfeld, Germany) of the same genetic background were used in age groups of 9–12 (young) or 37–42 (aged) weeks old. Male and female mice were included at random. Animals were socially housed in standard cages, exposed to a 12 h light/darkness cycle, and given ad libitum access to water and food. Where indicated, *Apoe*^−/−^ mice (10 weeks old) were fed with normal chow diet or with a cholate-free, high-fat diet (Arie Blok Diet W, Woerden, The Netherlands), consisting of (w/w) cocoa butter (15%), cholesterol (0.25%), sucrose (40.5%), corn starch (10%), corn oil (1%), cellulose (5.95%), casein (20%), 50% choline chloride (2%), methionine (0.2%) and mineral mixture (5.1%) with a total fat content of 16%^64,65^. For bone marrow transplantation, 1 × 10^7^ bone marrow cells obtained from wild type C57BL/6 J or *Ldlr*^−/−^ donor mice were injected into irradiated (2 × 6 Gy) *Ldlr*^−/−^ recipient mice^64^.

### Blood collection and processing

Mice were anaesthetized by subcutaneous injection of 100 mg/kg body weight ketamine and 0.5 mg/kg body weight medetomidine. Blood was taken through retro-orbital puncture, with 9 volumes collected into one volume of 129 mM trisodium citrate^66^, after which mice were euthanized by cervical dislocation. Where indicated, blood was collected into 300 µl PPACK/fragmin anticoagulant solution, containing saline, H-Phe-Pro-Arg chloromethyl ketone (PPACK, 40 μM), low molecular weight heparin (fragmin, 40 U/ml, Pfizer, Capelle a/d IJssel, The Netherlands) and heparin (5 units/ml, Sigma-Aldrich, St. Louis MO, USA)^67^. For the preparation of washed platelets, blood was collected 6:1 (vol./vol.) into acid citrate-dextrose solution (ACD, 80 mM sodium citrate, 52 mM citric acid, 183 mM *D*-glucose). Hematological blood parameters were determined in citrated blood using a Sysmex blood analyzer (Europe, Norderstedt, Germany). Platelet-free plasma (citrated) was stored at − 80 °C for later assessment of lipids and coagulation parameters.

### Real-time assessment of platelet–fibrin thrombus formation under flow

Our method to assess whole blood thrombus formation under coagulant conditions at defined wall shear rate^23^, was extended to a multiparameter assay^24^, and adapted for mouse blood. In brief, coverslips were coated with two separate surfaces. In the direction of flow: microspot *Ma*: collagen type I (2 μl, 50 μg/ml); and *Mb*: collagen type I + tissue factor (TF, 2 μl, 500 pM). After blocking with Tyrode's Hepes buffer pH 7.45 (5 mM Hepes, 136 NaCl, 2.7 mM KCl, 2 mM MgCl_2_, 0.42 NaH_2_PO_4_, 1 mg/mL glucose, 5 units/mL heparin) containing 1% BSA, the coverslips were mounted onto a parallel-plate flow chamber with precisely defined dimensions (depth 50 μm, width 3 mm and length 30 mm)^19^. Perfusion with recalcified citrated blood was performed until 6 min at a shear rate of 1000 s^−1^. Recalcification medium consisted of 63 mM CaCl_2_ and 32 mM MgCl_2_ in Tyrode's Hepes buffer; mixing with the blood was via a flattened Y-shaped dual inlet tube^23^. Before each experiment, blood samples (500 μl) were pre-labeled with DiOC_6_ (0.5 μg/mL, AnaSpec, San Jose CA, USA) and Alexa Fluor (AF) 647-labeled human fibrinogen (10 μg/mL, Molecular Probes, Bleiswijk, The Netherlands). Fluorescence (confocal) microscopic images were captured in two or three colors at 45 s intervals, to evaluate kinetics.

### Multiparameter assessment of platelet activation and aggregation under flow

Platelet adhesion and aggregation in perfused whole blood was measured under non-coagulant conditions as described^19,24^, but adapted for murine blood. Coverslips were coated with a microspot (0.5 µl) of collagen type I (100 µg/ml) and mounted onto a flow chamber, and perfused with PPACK/fragmin-anticoagulated blood for 3.5 min at a wall shear rate of 1000 s^−1^. Platelets were stained according to a stringent protocol by 2 min post-perfusion with Tyrode's Hepes buffer pH 7.45 (5 mM Hepes, 136 NaCl, 2.7 mM KCl, 2 mM MgCl_2_, 0.42 NaH_2_PO_4_, 2 mM CaCl_2_, 1 mg/mL glucose, 1 U/mL heparin, and 1 mg/mL BSA), supplemented with fluorescein isothiocyanate (FITC)-labeled rat anti-mouse CD62P mAb (1:40, Emfret Analytics, Würzburg, Germany), phycoerythrin (PE) labeled rat anti-mouse JON/A mAb (1:20, Emfret Analytics), and AF647 labeled annexin A5 (1:200, Invitrogen Life Technologies, Carlsbad, CA, USA). Representative end-stage brightfield microscopic images were taken from each microspot during staining. After 2 min of stasis, remaining labels were washed away by perfusion with label-free Tyrode's Hepes buffer, after which three representative end-stage fluorescence images were collected per microspot.

### Real-time microscopic image collection and quantitative image analysis

Microscopic images (1360 × 1024 pixels, 142 × 107 μm, 8-bit) were recorded with an EVOS fluorescence microscope (Life Technologies, Carlsbad CA, USA), equipped with GFP, RFP and Cy5 LED cubes, and an Olympus 60 × oil-immersion objective, basically as described^19^. For specific kinetic measurements, confocal fluorescence images were recorded with a LSM7 Live line-scanning system from Carl Zeiss (Jena, Germany)^23^.

Brightfield images, collected under non-coagulating conditions, were analyzed for the following parameters^19^: morphological score (*P1*, scale 0–5); percentage surface area coverage of deposited platelets (*P2*, % SAC), platelet aggregate contraction score (*P3*, scale 0–3), aggregate multilayer score (*P4*, scale 0–3), and aggregate multilayer coverage (*P5*, % SAC). Scores were compared against redefined standard images. Raw fluorescence images were analyzed for PS exposure (*P6*, % SAC), P-selectin expression (*P7*, % SAC) and integrin α_IIb_β_3_ activation (*P8*, % SAC). Images were examined by several observers. Two-color fluorescence images, collected under coagulating conditions, were analyzed for DiOC_6_ platelet deposition (*Pa*, % SAC); DiOC_6_ platelet thrombus score (P*b*, scale 0–5); time to first fibrin formation (*Pc,* − log min); AF647 fibrin score (*Pd*, scale 0–3) and AF647 fibrin-covered area (*Pe*, % SAC).

Values of SAC from brightfield and fluorescence images were determined, using specific scripts in Fiji software (Laboratory for Optical and Computational Instrumentation, University of Wisconsin-Madison WI, USA). Processing was as single color, gray images, either in 8-bit (brightfield) or in 24-bit (GFP, RFP or Cy5 fluorescence). Scripts opened series of one-color images using a loop. In each run, background illumination was corrected using a fast Fourier transform bandpass (FFTB) filter; this was followed by a threshold setting (with manual adjustment) and a surface area coverage measurement. For brightfield images, a series of Gray morphology conversions was applied to reduce striping and improve the detection (a diamond large-sized close, followed by a medium-sized circle close and a small circle-shaped dilate). The first step increased the pixels yet stronger in regions with many neighboring pixels, the second step rounded the shapes and additionally reduced straight lines, while the final step could be altered by user interface to match the overlap with unprocessed images. The FFTB filter sizes were selected to have minimal impact on the image structures, but flatten the background areas sufficiently for good analysis. For both brightfield and fluorescence channels, large structures were filtered down to 60–200 pixels, as appropriate per channel. Small structures were not filtered down, as these contained details of structures of interest within the platelets.

### Multiparameter data processing

For each flow run, parameter values from individual brightfield and fluorescence images were averaged to obtain one value per parameter per microspot. For the complete database of all runs, these values were linearly normalized to a scale from 0–10 per individual parameter. Gene effect heatmaps were obtained by subtracting per parameter the normalized average wild type values from the normalized average values of *Apoe*^−/−^ or *Ldlr*^−/−^ mice. Differences compared to wildtype were considered statistically significant with *P* < 0.05 (*t* test, 2-sided, equal variance) after correction for multiple comparisons, where required.

Effect sizes per parameter and genotype were calculated according to Cohen’s *d*: *d* = (*x͂*_1_ − *x͂*_2_)/*s*, in which the raw data per flow run were used to calculate the pooled standard deviation *s*, taking the equation: *s* = √(((*n*_1_-1) *s*_1_^2^ + (*n*_2_-1) *s*_2_^2^)/(*n*_1_ + *n*_2_-2)). Subsequently, regression coefficients *r* were determined by the equation *r* = *d*/√(*d*^2^ + *a*), in which *a* represents the correction factor if *n*_1_ ≠ *n*_2_, calculated by the equation: *a* = *(n*_1_ + *n*_2_*)*^2^*/n*_1_*n*_2_^68^. Normalized average and effect size heatmaps were produced using the R package version 3.2.5 (www.r-project.org).

### Flow cytometry

Flow cytometry with washed mouse platelets was performed with an Accuri C6 flow cytometer (Becton Dickinson, Ann Arbor MI, USA), as described^67^. Platelets (1 × 10^8^/mL) suspended in Tyrode's Hepes buffer pH 7.45 containing 2 mM CaCl_2_ were stimulated with convulxin (25–100 ng/mL)^69^, cross-linked collagen related peptide (CRP-XL, 0.5–5.0 μg/mL), methyl-thio-ADP (Me-S-ADP, 0.25–10 µM; Hart Biologicals, Hartlepool, UK), or thrombin (0.5–4.0 nM; Kordia, Leiden, The Netherlands), without stirring. Unstimulated samples were used as controls. After 15 min incubation, α-granule secretion was detected with FITC-labeled anti-CD62P mAb (1:10, Emfret Analytics, Wurzburg, Germany), integrin α_IIb_β_3_ activation with PE-labeled rat anti-mouse JON/A mAb (1:10, Emfret Analytics), and PS exposure with AF647-annexin A5 (1:200, Molecular Probes).

### Plasma lipids and coagulant activity

Platelet-free plasma was obtained by double centrifugation and stored at − 80 °C, as described before^70^. After thawing, plasma triglycerides were determined with a Triglycerides FS5 Ecoline kit from DiaSys (Diagnostic Systems, Holzheim, Germany); plasma cholesterol was measured with a Cholesterol FS10 kit from DiaSys. Thrombin-antithrombin (TAT) complexes in plasmas were measured with an Enzygnost TAT micro-kit from Siemens (Erlangen, Germany). Kits were used according to the manufacturer’s instructions.

### Calibrated automated thrombin generation

Thrombin generation with platelet-free plasma was performed using a 96 well plate-based assay, essentially following a published method^71^. Briefly, 20 μL of murine plasma was mixed with 10 μL of pre-trigger medium containing 7 pM tissue factor (Dade Innovin, Siemens, the Hague, The Netherlands), 0.83 mg/mL BSA, and 60 μM phospholipid mixture (PS/PE/PC, 20/60/20, w/w/w) in 25 mM Hepes plus 165 mM NaCl (pH 7.5). Mixtures were incubated at 37 °C for 7 min, and then supplemented with recalcification buffer containing chromogenic thrombin substrate or α_2M_-thrombin calibrator^71^. Thrombin generation was immediately calculated per well using Thrombinoscope software (Maastricht, The Netherlands).

### Preparation of highly purified platelets for proteomics or lipidomics

Sample preparation for proteomic and lipidomic analysis was performed, as described for human blood^29^, with adjustments for mouse blood to maximize purified platelet yield. In brief, platelets for proteomics were prepared from ACD-anticoagulated blood from one animal in an Eppendorf tube, which was supplemented with EGTA (0.5 mM) and apyrase (0.2 U/mL). Platelets for lipidomic analysis were prepared from citrate-anticoagulated blood. After centrifugation at 264* g* for 5 min, the platelet-rich plasma (PRP) with the top 30% of red blood cells was transferred to a new tube. PRP was obtained by centrifugation at 80* g* for 6 min, and transferred to a new tube. After addition of 200 μL Tyrode's Hepes buffer (137 mM NaCl, 2 mM KCl, 12 mM NaHCO_3_, 0.3 mM NaH_2_PO_4_, 5.5 mM glucose, 5 mM Hepes, pH 7.4) to the remaining 30% red blood cells, a second lot of PRP was obtained by another centrifugation step as above. The combined PRP samples were supplemented with ACD and apyrase, and centrifuged at 720* g* for 6 min. The pelleted platelets were carefully resuspended into an adequate volume of Tyrode's Hepes buffer containing ACD (1:10) and apyrase (1 U/mL), leaving the red cell pellet untouched, transferred to a clean Eppendorf tube and centrifuged again. This procedure was repeated, if necessary, to remove residual red and white blood cells.

For proteomic analyses platelets were isolated from 4 wild-type, 4 *Apoe*^−/−^ and 3 *Ldlr*^−/−^ mice, and the final platelet pellet was resuspended in 150 µL Tyrode's Hepes buffer. Purity of the platelet suspension was verified using a Sysmex blood analyzer and by microscopic analysis, and approved when contamination of red cells and leukocytes was < 1:15,000 and < 1:20,000, respectively, in agreement with previously published data^12^. The pure platelet samples were lysed with lysis buffer 1:1 pH 7.8 (50 mM Tris, 1% SDS, 150 mM NaCl, 1 tablet PhosStop/7 mL; Roche, Basel, Switzerland) containing a mixture of protease and phosphatase inhibitors (10 mM sodium orthovanadate (Na_3_VO_4_, Sigma-Aldrich, St. Louis MO, USA) 4 mM phenylmethylsulfonyl fluoride (ThermoFisher Scientific, Breda, The Netherlands), 20 μg/mL leupeptin (VWR, Amsterdam, The Netherlands), 5 μg/mL pepstatin (VWR, Amsterdam, The Netherlands) and 4 mM EDTA). Lysed samples were immediately snapfrozen and stored at − 80 °C until use.

For lipidomic analysis the final platelet pellet and the supernatant were separated and immediately snapfrozen and stored at − 80 °C until usage for lipidomics analysis, which was performed basically as described^59^. During the procedures, blank extractions were performed for all analysis to detect possible contamination which could lead to false positive identification. Platelets in the analyzed preparations were unstimulated, verified by the low amounts of secreted 12-HETE (all samples below 400 pmol/mg protein) and thromboxane B_2_ (all below 45 pmol/mg protein).

### Proteomic analysis

A label-free quantification analysis was performed with mouse platelet samples, as described before^29^. In short, the protein concentration of well-purified platelet samples (277–1390 × 10^9^/L) in 1% SDS lysis buffer was estimated using the Bicinchoninic acid assay kit (Pierce, Thermo-Fisher Scientific, Bremen, Germany). After equalizing protein concentration, proteins were reduced with dithiothreitol and free sulfhydryl groups were alkylated with iodoacetamide. Subsequently, 20 µg of protein sample were processed using filter-aided sample preparation (FASP), with a 30 kDa molecular weight cut-off spin filter. Thus, digestion was conducted with 50 mM ammonium bicarbonate, 200 mM guanidinium hydrochloride and 2 mM CaCl2, pH 7.8 in the presence of trypsin (1:20 w/w, sequencing grade trypsin Promega, Madison WI, USA) for 14 h at 37 °C. Trypsin digestion was monitored by monolithic reversed phase HPLC^72^. For label free analysis 2 µg of peptide, were analyzed by nano LC–MS/MS in DDA mode using a U3000 nano-RSLC system online-coupled to an Orbitrap Lumos mass spectrometer. Thus, peptides were loaded onto a trap column (Acclaim PepMap 100 C18; 100 μm × 2 cm) with 0.1% trifluoroacetic acid (TFA). Then, peptides were separated on the main column (PepMap 100 C18; 75 μm × 50 cm), using a binary gradient ranging from 3–45% solvent B (84% acetonitrile, 0.1% formic acid) in 150 min at 60 °C and a flow rate of 250 nL/min. In the Lumos Orbitrap survey scans were acquired in the Orbitrap at resolution of 120,000 FWHM, with an AGC target of 2 × 105 and a maximum injection time of 50 ms. Data dependent MS/MS was performed using Top speed parameter with 3 s cycle time of the most intense signals using monoisotopic peak determination and an intensity threshold of 5 × 10^3^, a dynamic exclusion of 15 s and an isolation window of 1.2 m/z using the quadrupole. Thus, peptide fragments were acquired in the ion trap in rapid scan mode with a first fixed mass of 120 m/z after fragmentation by Higher collision induced dissociation with 30% normalized collision energy. Additionally, an automatic gain control target value of 2 × 10^3^ and maximum injection time of 300 ms with Inject ion for all available parallelizable time were used.

For label free quantification the Progenesis LC–MS software version 4.1 from Nonlinear Dynamics (Newcastle upon Tyne, UK) was used. Consequently, MS raw data were aligned in automatic mode. Exported peak lists were searched using SearchGUI 3.3.3, Mascot 2.6 (Matrix Science), X! TANDEM Vengeance (2015.12.15.2) and MS-GF + Release (v2018.04.09), with a concatenated target/decoy version of the mouse Uniprot database (July 2015, containing 16,716 sequences). Data analysis was done using PeptideShaker 1.16.23 (http://code.google.com/p/peptide-shaker/), in order to maximize number of identified peptides and proteins. The following search parameters were used: trypsin as protease with a maximum of two missed cleavages; carbamidomethylation of Cys (57.021464) as fixed and as variable modification oxidation of Met (15.994915), acetylation of protein N-term and acetylation of K (42.010565). MS and MS/MS tolerances were set to 10 ppm and 0.5 Da. In PeptideShaker, search results were combined and filtered at a FDR of 1% (high confidence filter) on the protein level prior to export and re-import into Progenesis. Peptide sequences containing pyro-Glu (derived from X!Tandem 2nd pass search), oxidized methionine and acetylation of lysine and protein N-terminal were omitted from further data analysis. Only proteins with at least two unique peptides were considered for quantification. The mass spectrometry proteomics data have been deposited to the ProteomeXchange Consortium via the PRIDE^73^ partner repository with the dataset identifier PXD020276. For comparison between platelets from both knockout against wild-type mice described common platelet contaminant proteins^53^ were removed and raw values were normalized. After normalization, a log 2 ratio was calculated, as well as *P* values (*t* test). In total, 1,533 proteins were quantified and regulated proteins were defined as 1.5-fold up- or downregulated.

### Statistics

Data are presented as means ± SEM. Comparisons between age, diet and mouse genotypes (knock-out compared to wild-type) were made using the Mann Whitney U-test or the Kruskal–Wallis test for continuous variables. As we have shown previously, the majority of parameters tended to correlate positively per microspot, suggesting a 'thrombus profile' represented by multiple parameters^24^. Therefore, datasets were compared using Pearson or Spearman correlation analysis, as described^24^.

For proteomic analysis, a t-test was used after normalization to compare up-or downregulated proteins between wild-type and knock-out mice. As multiple hypotheses were tested simultaneously, we corrected for multiple testing and controlled the false discovery rate (FDR; 0.05) with the Benjamini–Hochberg procedure, which adapts to the amount of signal in the data. The statistical package for social science version 22 was used (SPSS 22, Chicago IL, USA), with a *P* value < 0.05 considered to be significant.

## Supplementary information

Supplementary Figure 1.

Supplementary Figure 2.

Supplementary Figure 3.

Supplementary Figure 4.

Supplementary Information 1.

Supplementary Information 2.
