# Supplementary figures and images for: Mild hyperlipidemia in mice aggravates platelet responsiveness in thrombus formation and exploration of platelet proteome and lipidome
Source: Sci Rep. 2020 Dec 8;10:21407. doi: 10.1038/s41598-020-78522-9 (PMC7722935; doi:10.1038/s41598-020-78522-9)

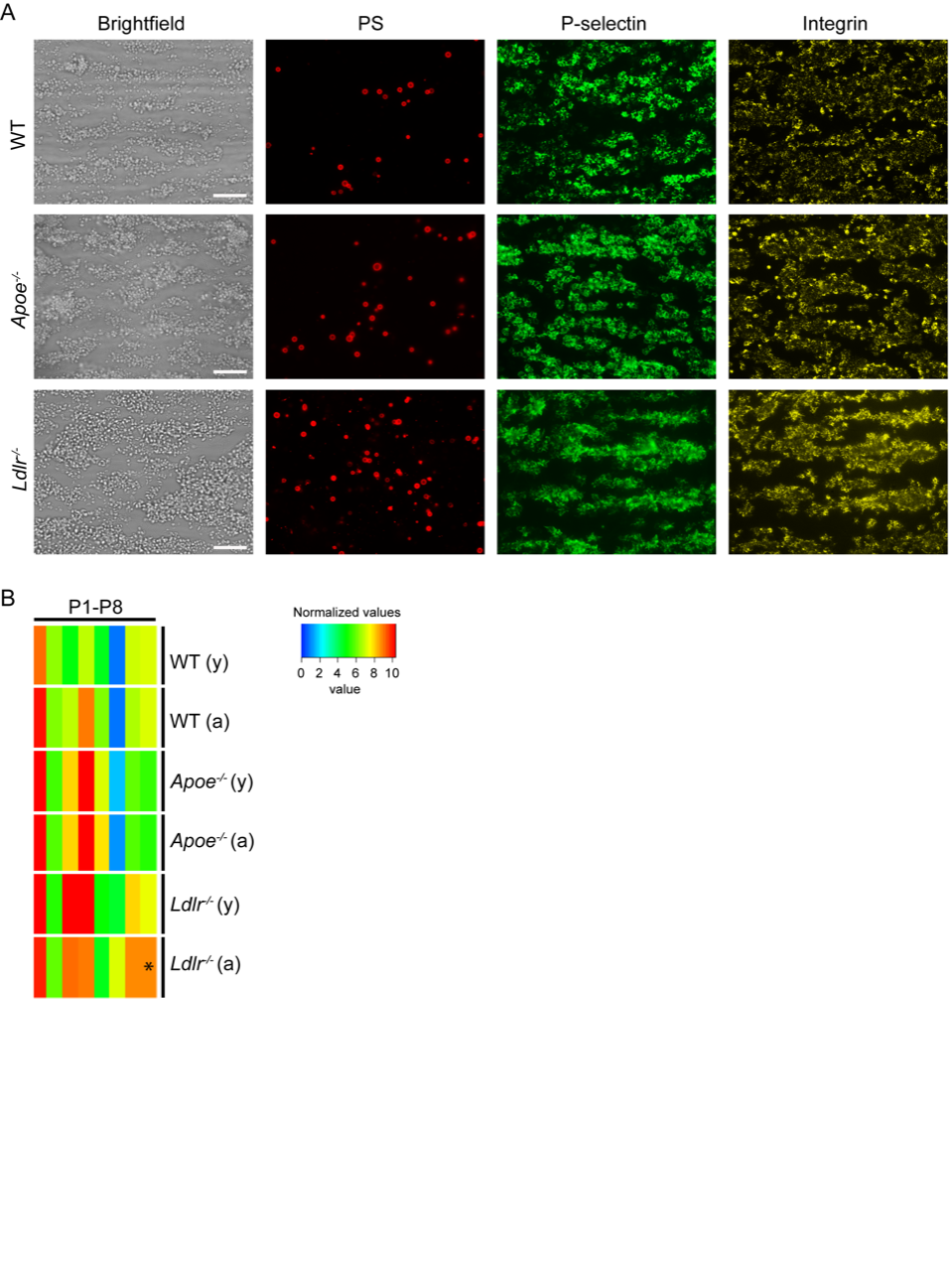

Supplement: Supplementary file 1 — Supplementary Figure 1. [file 41598_2020_78522_MOESM1_ESM.tiff]

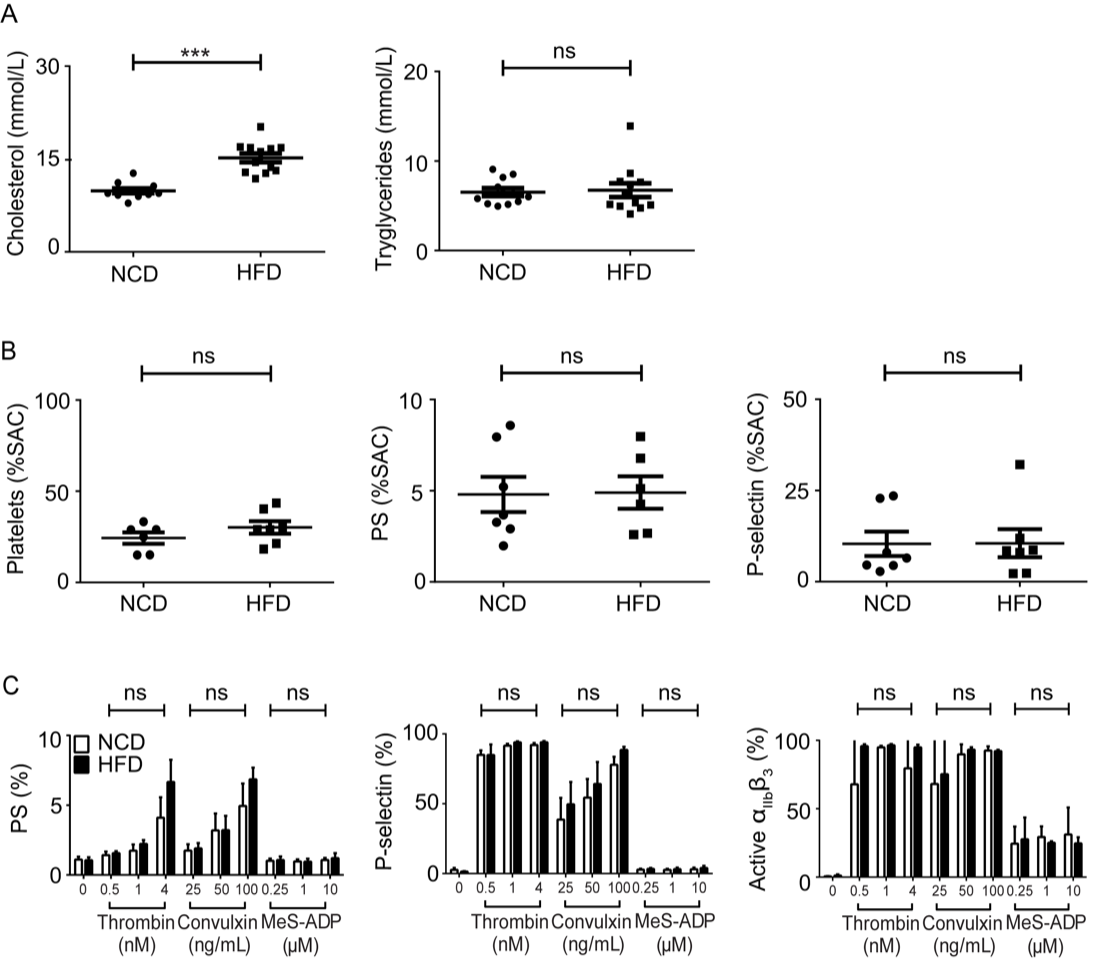

Supplement: Supplementary file 2 — Supplementary Figure 2. [file 41598_2020_78522_MOESM2_ESM.tiff]

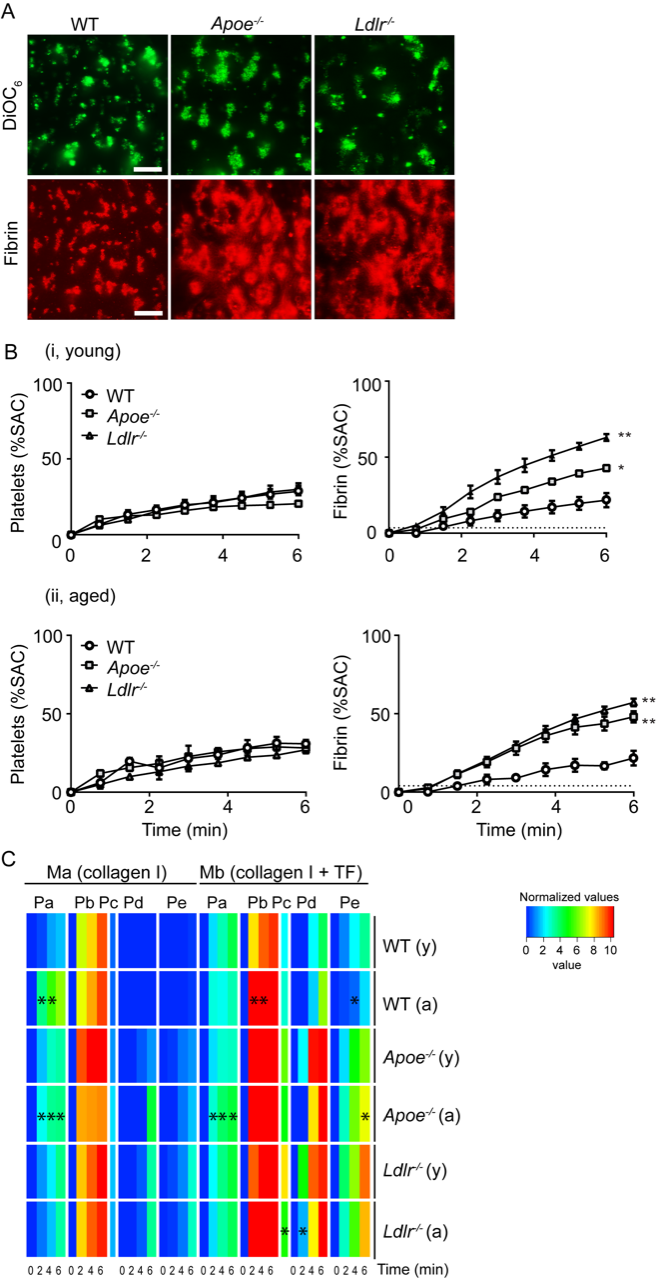

Supplement: Supplementary file 3 — Supplementary Figure 3. [file 41598_2020_78522_MOESM3_ESM.tiff]

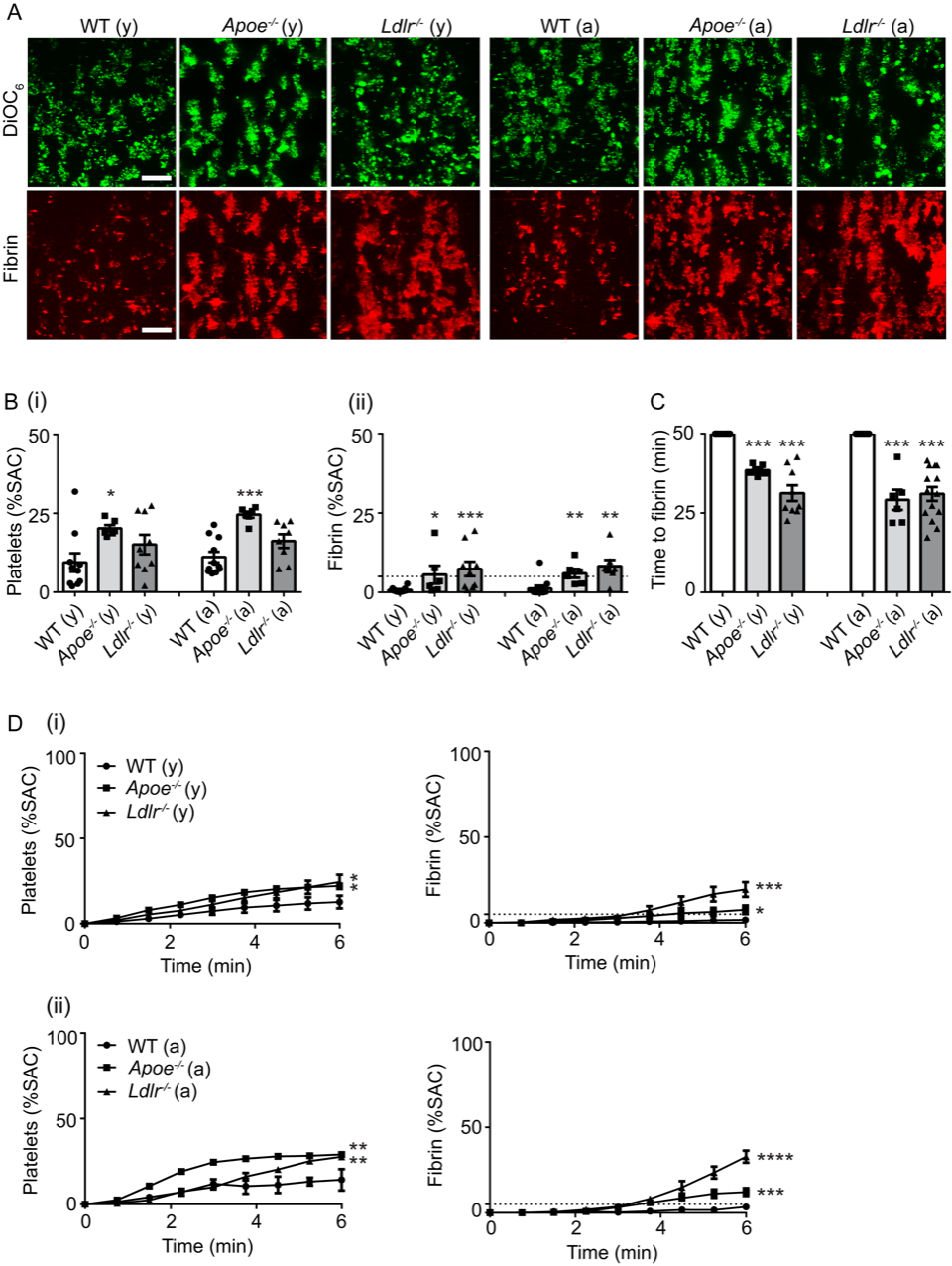

Supplement: Supplementary file 4 — Supplementary Figure 4. [file 41598_2020_78522_MOESM4_ESM.tiff]
