# Supplementary material for: Mild hyperlipidemia in mice aggravates platelet responsiveness in thrombus formation and exploration of platelet proteome and lipidome
Source: Sci Rep. 2020 Dec 8;10:21407. doi: 10.1038/s41598-020-78522-9 (PMC7722935; doi:10.1038/s41598-020-78522-9)
Supplement: Supplementary file 5 — Supplementary Information 1. [file 41598_2020_78522_MOESM5_ESM.docx]

**Supplementary Material**

**Mild hyperlipidemia in mice aggravates platelet responsiveness in thrombus formation and exploration of platelet proteome and lipidome.**

Johanna P. van Geffen, Frauke Swieringa, Kim van Kuijk, Bibian M.E. Tullemans, Fiorella A. Solari, Bing Peng, Kenneth J. Clemetson, Richard W. Farndale, Ludwig J. Dubois, Albert Sickmann, René P. Zahedi, Robert Ahrends, Erik A. L. Biessen, Judith C. Sluimer, Johan W. M. Heemskerk, Marijke J. E. Kuijpers.

**Suppl. Table 1. Blood parameters and lipid levels of young and aged wild-type, *Apoe^-/-^* and *Ldlr^-/-^* mice.** Hematological parameters

assessed in citrated blood from young (9-12 weeks) or aged (37-42 weeks) wild-type (C57BL/6J), *Apoe^-/-^* and *Ldlr^-/-^* mice, held on

normal chow diet. Cholesterol and triglyceride levels were assessed in platelet-free plasma. Means ± SEM (*n*=6-14), **P*<0.05

*vs*. wild-type, ^#^*P*<0.05 *vs.* young (t-test). Green (red) represents significantly lower (higher) values.

| **Parameter** | **Wild-type** |  | ***Apoe^-/-^*** |  | ***Ldlr^-/-^*** |  | |
| --- | --- | --- | --- | --- | --- | --- | --- |
|  | young | aged | young | aged | young | aged | |
| Platelet count x10^9^, l^-1^ | 706 ± 34 | 1045 ± 199^#^ | 819 ± 68 | 1020 ± 105^#^ | 459 ± 49 | 689 | ± 85^#^* |
| Mean platelet volume, fl | 5.68 ± 0.05 | 6.05 ± 0.09^#^ | 5.84 ± 0.06 | 5.83 ± 0.03 | 6.08 ± 0.25 | 6.08 ± 0.06 | |
| Hematocrit, l/l | 37.92 ± 1.87 | 38.25 ± 1.03 | 41.43 ± 0.87 | 40.33 ± 0.88 | 33.83 ± 1.62 | 35.91 ± 1.52 | |
| Leukocyte count x 10^9^, l^-1^ | 4.42 ± 0.34 | 6.25 ± 0.25^#^ | 3.53 ± 0.94 | 4.33 ± 0.33 | 4.83 ± 0.46 | 4.59 ± 0.35 | |
| Cholesterol, mM | 1.58 ± 0.40 | 1.80 ± 0.31 | 5.58 ± 0.64* | 11.86 ± 0.99*^#^ | 3.50 ± 0.41* | 5.50 ± 1.36* | |
| Triglycerides, mM | 2.77 ± 0.45 | 2.25 ± 0.077 | 2.87 ± 0.67 | 4.85 ± 0.84 | 3.38 ± 0.22 | 2.41 ± 1.25 | |

**Suppl. Table 2. Blood parameters and lipid levels of bone marrow transplantation mouse.** Hematological parameters assessed in citrated blood from *Ldlr^-/-^* mice transplanted with bone marrow from wild-type *(WT^BM^)* or *Ldlr^-/-^* mice *(Ldlr^-/-BM^*), held on normal chow diet. Cholesterol levels were assessed in platelet-free plasma. Means ± SEM (*n* = 6). ^*^*P*<0.05 (Mann-Whitney U test).

| **Parameter** | *WT^BM^* | *Ldlr^-/-BM^* |
| --- | --- | --- |
| Platelet count x10^9^, l^-1^ | 412 ± 75.26 | 532 ± 92.92 |
| Mean platelet volume, fl | 6.36 ± 0.14 | 6.16 ± 0.09 |
| Hematocrit, l/l | 35.80 ± 1.50 | 35.20 ± 1.16 |
| Leukocyte count x 10^9^, l^-1^ | 5.4 ± 0.40 | 6.6 ± 0.24 |
| Cholesterol, mM | 3.61 ± 0.69 | 3.43 ± 0.55 |

**Suppl. Table 3. Changes in quantitative platelet proteome of *Apoe* and *Ldlr* deficient mice.** Separately uploaded excel file. Highly purified platelets from wild-type, *Apoe^-/-^* and *Ldlr^-/-^* mice were subjected to label-free proteomics analysis. Ratios of 1,533 unique proteins were quantified (see Methods). Median peptide ratios per protein were log2 transformed. Listed proteins with largest differences in abundance levels (1.5-fold changed expression) for **A**) *Apoe^-/-^* and **B**) *Ldlr^-/-^* mice. **C**) Platelet proteins that are significantly changed in expression (at least 1.5-fold) in both knockout mice (t-test). Red color represents upregulated proteins and green color downregulated proteins (-1: downregulated, 1: upregulated, 0: below 2-fold change in expression). *n* = 3-4 animals/group.

**Supplemental Table 4: Described effects on platelet function of significantly altered platelet proteins from *Apoe^-/-^* and *Ldlr^-/-^* mice.** Literature overview of effects on platelet function of significantly altered platelet proteins as detected by proteomic analysis.

| **Name protein** | **Changed in *Apoe^-/-^* or *Ldlr^-/-^*** | **Up- or down regulated** | **Described effect on platelet function or coagulation** | **Results of functional assays** | **Ref** |
| --- | --- | --- | --- | --- | --- |
| Thrombospondin-2 | Both | Up | TSP-2 is required for normal MK function and platelet formation.  TSP-2 deficient mice are impaired in vWF adhesion and platelet responses. | LTA: TSP-2 deficient mice display suboptimal ADP-induced aggregation, while aggregation in response to collagen and thrombin is normal.  Flow chamber experiments: reduced VWF accumulation on TSP-2 deficient ECM was observed resulting in reduced thrombus formation.  In vivo: TSP-2 deficient mice show a bleeding phenotype and are protected from arterial thrombosis. | ^1,2^ |
| Semaphorin-4B | Both | Up | Unknown role in platelet function, but semaphorin 3A, 4D and 7A have been shown to play a role in platelet function. | The role of semaphorin-4B in platelet function has not been investigated. | ^3,4^ |
| Complement factors | Both  *Apoe^-/-^*: C3 and C4-B  *Ldlr^-/-^*: B | Up | Complement C3 and complement C4 precursor proteins are secreted from platelet α-granules. Complement-induced platelet activation, shedding of extracellular vesicles, and thrombo-inflammation. | Flow cytometry: C3 binds directly to (thrombin-)activated platelets via P-Selectin.  Flow chamber experiments: TRAP-activated platelets formed aggregates on a C3b coated surface under flow, which was dependent on P-selectin. C4 activation appears particularly enhanced following platelet exposure to shear stress.  In vivo: Mice lacking C3 show prolonged bleeding times and reduced thrombus and fibrin formation in venous thrombosis. | ^5-9^ |
| Alpha-1-antitrypsin | Both  *Apoe^-/-^*: 1-5 *Ldlr^-/-^*: 1-4 | Up | Inhibition of plasma proteases such as APC, which may result in higher thrombin levels in plasma. | In vivo: complex formation with APC in baboons | ^10,11^ |
| Factor XIII | *Apoe^-/-^* | Up | Fibrin polymerization. Stimulation of coated platelet formation. | Flow cytometry and flow chamber experiments: A subpopulation of phosphatidylserine-exposing platelets bound Factor XIII. In suspension, high fibrinogen and Factor XIII binding were antagonized by combined inhibition of transglutaminase activity and integrin α(IIb)β3.  In vivo: FeCl3-induced thrombus formation is unchanged in FXIII-deficient mice. | ^12-15^ |
| Reticulon-4 (Nogo) | *Apoe^-/-^* | Up | Involved in formation of procoagulant platelets, possibly via store-operated calcium entry. | Platelet spreading on fibrinogen: p38-MAPK2-RTN4-Bcl-xl pathway associated with the regulation of the ER and platelet phosphatidylserine exposure.  Mouse embryonic fibroblast cell line: reticulon-4 is required for STIM1-Orai1 coupling, and store-operated calcium entry. | ^16,17^ |
| Amyloid beta A4 protein | *Apoe^-/-^* | Up | Potentiates thrombus formation via binding to integrin αIIbβ3, and release of ADP and clusterin. | Flow cytometry: Increasing concentrations of Aβ induced binding of fibrinogen.  LTA: Aβ induces platelet aggregation  Static adhesion and adhesion under flow: platelets adhere to immobilized Aβ.  Western blotting: platelet stimulation with Aβ results in phosphorylation of Syk and PLCγ2. | ^18,19^ |
| Clusterin | *Apoe^-/-^* | Up | Chaperone involved in protein folding of secreted proteins, and involved in lipid transport. Also connected to amyloid-β aggregation via integrin αIIbβ3. Binds and inhibits complement factors. | ELISA and western blotting: platelets secrete increased amounts of clusterin in response to Aβ, which was reduced by blocking integrin αIIbβ3. | ^9,18^ |
| Integrin-linked protein kinase | *Apoe^-/-^* | Down | ILK regulates integrin function and alpha-granule secretion, and is essential for stable thrombus formation. | LTA: ILK-deficient platelets and human platelets inhibited with QLT 0267 (1 μm ) show reduced collagen-induced aggregation.  Flow cytometry: ILK-deficient platelets show reduced fibrinogen binding upon stimulation with collagen and thrombin. P-selectin expression is reduced upon collagen stimulation.  Flow chamber experiments: thrombus formation on collagen under arterial flow conditions is reduced in ILK-deficient mice.  In vivo: ILK-deficient mice showed an increased bleeding time and volume. Upon laser injury of the cremaster arteriole thrombus size was unaffected in ILK-deficient mice, but thrombus stability was markedly decreased. | ^20,21^ |
| Rab- and Rap-GTP-ases (several: suppl. Table 3) | *Apoe^-/-^* | Down | Key regulators of intracellular membrane trafficking. | Many functional assays in platelets as reviewed in ^22^. E.g. IP and intracellular calcium: thrombin and convulxin (GPVI-agonist) activate Rap2B, which is regulated by intracellular calcium. | ^22,23^ |
| Protein kinases  (several: suppl. Table 3) | *Apoe^-/-^* | Both | Essential for platelet signaling under multiple receptors. | Many functional assays in platelets as reviewed in ^24,25^. | ^24,25^ |
| Protein phosphatases  (several: suppl. Table 3) | *Apoe^-/-^* | Both | Essential for platelet signaling under multiple receptors. | Many functional assays in platelets as reviewed in ^26^. E.g. western blotting: inhibition of PP2Ac in resulted in Src activation and its downstream ERK1/2 signaling pathways that regulate αIIbβ3 adhesion. | ^26,27^ |
| Tetraspanin-9 (CD9) and -13 | *Apoe^-/-^* | 9: down  13: up | Fine tuning of platelet responses via regulation of other proteins in microdomains.  9: Negatively regulates integrin αIIbβ3 activation, and associates with CD36. | IP and confocal microscopy: CD9 associates with CD36  Flow cytometry: normal P-selectin expression, but increased fibrinogen binding of CD9-deficient platelets in response to several agonists.  Flow chamber experiments: thrombus formation on collagen was unchanged in CD9-deficient mice under high and low shear conditions.  In vivo: CD9-deficient mice showed normal bleeding time. FeCl3-induced thrombus formation at the carotid artery showed larger thrombi in CD9-deficient mice at later stages.  The role of tetraspanin-13 in platelet function has not been investigated. | ^28-30^ |
| Glycoprotein Ibα | *Ldlr^-/-^* | Up | Receptor of vWF, critical for platelet adhesion under high shear conditions. Receptor for thrombin and other coagulation factors, thrombospondin-1 and P-selectin. | Flow chamber experiments: platelet tethering on (collagen-bound) vWF is dependent on GPIb under high shear conditions.  In vivo: GPIb-deficient mice are protected from arterial thrombosis and display a bleeding phenotype. | ^31-34^ |
| Vitamin-D-binding protein | *Ldlr^-/-^* | Up | Lowers vitamin-D levels in blood, associated with higher platelet reactivity. Acts as an actin scavenger in plasma. | Multiplate: lower vitamin D levels were associated with higher platelet reactivity and impaired effectiveness of the ADP-antagonists clopidogrel and ticagrelor. | ^35^ |

Abbreviations: IP: immunoprecipitation, MK: megakaryocytes, LTA: Light transmission aggregometry, vWF: von Willebrand Factor

**Suppl. Figure 1. Multiparameter assessment of platelet activation and aggregation in the absence of coagulation under flow of aged *Apoe^-/-^* and *Ldlr^-/-^* mice *ex vivo*.** *Apoe^-/-^*, *Ldlr^-/-^* and corresponding wild type mice (37-42 weeks old) were held on normal chow diet. Blood was anticoagulated with PPACK/fragmin/heparin and was perfused over collagen type I at a shear rate of 1000 s^-1^ for 3.5 min. Brightfield images were captured, after which the deposited platelets were stained for integrin α_IIb_β_3_ activation, P-selectin expression and PS exposure in different colors (see Methods). **A**) Representative images are shown. Bars indicate 20 μm. **B**) Platelet activation parameters were obtained from brightfield and fluorescence images after 3.5 min: *P1*, morphological score; *P2*, platelet surface area coverage (% SAC); *P3*, aggregate contraction score; *P4*, aggregate multilayer score; *P5*, aggregate multilayer coverage (% SAC); *P6*, PS exposure (% SAC); *P7*, P-selectin expression (% SAC); *P8*, integrin α_IIb_β_3_ activation (% SAC). Values per parameter were linearly scaled to 0-10. Shown is a scaled heatmap of mean flow runs per age group (young (y, 9-12 weeks old) or aged (a, 37-42 weeks old)) and genotype. * *P*<0.05 of age groups per genotype (Mann-Whitney U test).

**Suppl. Figure 2. Effect of high fat diet on activation of *Apoe^-/-^* platelets.** *Apoe^-/-^* mice, aged 18 weeks, were fed during 10 weeks normal chow diet (NCD) or high fat diet (HFD). **A**) Levels of plasma cholesterol and triglycerides after 10 weeks of diet. **B**) Brightfield and fluorescence images were taken after 3.5 min of perfusion at 1000 s^-1^ of PPACK-anticoagulated whole blood over collagen type I microspots. See further, Figure 1. Means ± SEM (*n* = 11-12 animals/group). **C**) Washed platelets were activated with thrombin (0.5-4 nM), the GPVI agonist convulxin (25-100 ng/mL), or Me-S-ADP (0.25-10 µM). Using flow cytometry, exposure of phosphatidylserine (PS) was determined with AF647-labeled annexin A5, P-selectin expression with FITC-labeled anti-CD62P mAb, and activation of integrin α_IIb_β_3_ with PE-labeled JON/A mAb. Means ± SEM (*n* = 6 animals/group).

**Suppl. Figure 3**. **Deficiency in ApoE or LDLR enhances formation of platelet-fibrin thrombi in the presence of coagulation at arterial flow rate in mice.** Blood was obtained from young (9-12 weeks) and aged (37-42 weeks) *Apoe^-/-^*, *Ldlr^-/-^* and corresponding wild-type mice held on normal chow diet. Citrated blood samples were supplemented with DiOC_6_ (labeling platelets) plus AF647 fibrinogen, and co-perfused with CaCl_2_/MgCl_2_ over two microspots consisting of collagen (microspot *Ma*) and collagen/TF (microspot *Mb*) at a wall shear rate of 1000 s^-1^. Confocal two-color fluorescence images were captured in real time at 45 s time intervals. **A**) Representative images from aged mice of deposited platelets (DiOC_6_, green) and fibrin (AF647, red) at spot *Mb* after 4 min; bars = 20 µm. **B**) Kinetic quantification of platelet deposition and fibrin-covered area per genotype of (i) young and (ii) aged mice. Horizontal dotted line indicates labeling threshold for fibrin formation. Means ± SEM (*n* = 6-14 animals/group). **P*<0.05, **P<0.01, ***P<0.001 *vs.* wild-type (Mann-Whitney U test). **C)** Thrombus parameters were obtained from brightfield and fluorescence images at time points 0, 2, 4 and 6 min: *Pa*, DiOC_6_ platelet deposition (surface area coverage, % SAC); *Pb*, DiOC_6_ platelet thrombus score (0-5); *Pc*, time to first fibrin formation (-log min); *Pd*, AF647 fibrin score; *Pe*, AF647 fibrin-covered area (% SAC). Values per parameter were linearly scaled to 0-10. Shown is a scaled heatmap of mean flow runs per age group (young (y) and aged (a)) and genotype. * *P*<0.05 between age groups per genotype (Mann-Whitney U test).

**Suppl. Figure 4**. **Deficiency in ApoE or LDLR enhances formation of platelet-fibrin thrombi at arterial flow rate ex vivo in the absence of tissue factor.** Blood was obtained from *Apoe^-/-^*, *Ldlr^-/-^* and corresponding wild type mice held on normal chow diet; animals were young (y, 9-12 weeks old) or aged (a, 37-42 weeks old). Citrated blood samples were supplemented with DiOC_6_ (labeling platelets) plus AF-647 fibrinogen, and co-perfused with CaCl_2_/MgCl_2_ over two microspots consisting of collagen (microspot *Ma*) and collagen/TF (microspot *Mb*) at a wall-shear rate of 1000 s^-1^. Confocal two-color fluorescence images were captured in real time at 45 s time intervals. **A**) Representative images of deposited platelets (DiOC_6_, green) and fibrin (AF-647, red) at spot *Ma* after 4 min; bars = 20 µm. **B**) Quantification of surface area coverage (% SAC) of platelet deposition (*i*), and fibrin-covered area (*ii*) at spot *Ma* after 4 min; horizontal dotted line indicates labeling threshold for fibrin formation. **C**) Kinetic quantification of platelet deposition and fibrin-covered area per age group (*i*; young, *ii*; aged), per genotype. Horizontal dotted line indicates labeling threshold for fibrin formation. Means ± SEM (*n* = 6-14 animals/group). **P*<0.05, **P<0.01, ***P<0.001 *vs.* wild-type (Mann-Whitney U test).

**References**

1 Kyriakides, T. R. *et al.* Megakaryocytes require thrombospondin-2 for normal platelet formation and function. *Blood* **101**, 3915-3923 (2003).

2 Kristofik, N. *et al.* Impaired von Willebrand factor adhesion and platelet response in thrombospondin-2 knockout mice. *Blood* **128**, 1642-1650 (2016).

3 Wannemacher, K. M. *et al.* An expanded role for semaphorin 4D in platelets includes contact-dependent amplification of Clec-2 signaling. *J. Thromb. Haemost.***11**, 2190-2193 (2013).

4 Wannemacher, K. M., Wang, L., Zhu, L. & Brass, L. F. The role of semaphorins and their receptors in platelets: Lessons learned from neuronal and immune synapses. *Platelets* **22**, 461-465 (2011).

5 Karpman, D. *et al.* Complement Interactions with Blood Cells, Endothelial Cells and Microvesicles in Thrombotic and Inflammatory Conditions. *Adv. Exp. Med. Biol.* **865**, 19-42 (2015).

6 Nording, H. & Langer, H. F. Complement links platelets to innate immunity. *Semin. Immunol.* **37**, 43-52 (2018).

7 Subramaniam, S. *et al.* Distinct contributions of complement factors to platelet activation and fibrin formation in venous thrombus development. *Blood* **129**, 2291-2302 (2017).

8 Del Conde, I., Cruz, M. A., Zhang, H., Lopez, J. A. & Afshar-Kharghan, V. Platelet activation leads to activation and propagation of the complement system. *J. Exp. Med.* **201**, 871-879 (2005).

9 Peerschke, E. I., Yin, W. & Ghebrehiwet, B. Platelet mediated complement activation. *Adv. Exp. Med. Biol.* **632**, 81-91 (2008).

10 van der Meer, F. J. *et al.* A second plasma inhibitor of activated protein C: alpha 1-antitrypsin. *Thromb. Haemost.* **62**, 756-762 (1989).

11 Espana, F. *et al.* In vivo and in vitro complexes of activated protein C with two inhibitors in baboons. *Blood* **77**, 1754-1760 (1991).

12 Mattheij, N. J. *et al.* Coated platelets function in platelet-dependent fibrin formation via integrin alphaIIbbeta3 and transglutaminase factor XIII. *Haematologica* **101**, 427-436 (2016).

13 Dale, G. L. *et al.* Stimulated platelets use serotonin to enhance their retention of procoagulant proteins on the cell surface. *Nature* **415**, 175-179 (2002).

14 Mitchell, J. L. & Mutch, N. J. Novel aspects of platelet factor XIII function. *Thromb. Res.* **141 Suppl 2**, S17-21 (2016).

15 Tang, Z. *et al.* Factor XIII deficiency does not prevent FeCl3-induced carotid artery thrombus formation in mice. *Res. Pract. Thromb. Haemost.* **4**, 111-116 (2020).

16 Babur, O. *et al.* Platelet procoagulant phenotype is modulated by a p38-MK2 axis that regulates RTN4/Nogo proximal to the endoplasmic reticulum: utility of pathway analysis. *Am. J. Physiol. Cell. Physiol.* **314**, C603-C615 (2018).

17 Jozsef, L. *et al.* Reticulon 4 is necessary for endoplasmic reticulum tubulation, STIM1-Orai1 coupling, and store-operated calcium entry. *J. Biol. Chem.* **289**, 9380-9395 (2014).

18 Donner, L. *et al.* Platelets contribute to amyloid-beta aggregation in cerebral vessels through integrin alphaIIbbeta3-induced outside-in signaling and clusterin release. *Sci. Signal.* **9**, ra52 (2016).

19 Visconte, C. *et al.* Amyloid precursor protein is required for in vitro platelet adhesion to amyloid peptides and potentiation of thrombus formation. *Cell. Signal.* **52**, 95-102 (2018).

20 Tucker, K. L. *et al.* A dual role for integrin-linked kinase in platelets: regulating integrin function and alpha-granule secretion. *Blood* **112**, 4523-4531 (2008).

21 Jones, C. I. *et al.* Integrin-linked kinase regulates the rate of platelet activation and is essential for the formation of stable thrombi. *J. Thromb. Haemost.* **12**, 1342-1352 (2014).

22 Walsh, T. G., Li, Y., Wersall, A. & Poole, A. W. Small GTPases in platelet membrane trafficking. *Platelets*, 1-10 (2018).

23 Greco, F., Sinigaglia, F., Balduini, C. & Torti, M. Activation of the small GTPase Rap2B in agonist-stimulated human platelets. *J. Thromb. Haemost.* **2**, 2223-2230 (2004).

24 Watson, S. P., Auger, J. M., McCarty, O. J. & Pearce, A. C. GPVI and integrin alphaIIb beta3 signaling in platelets. *J. Thromb. Haemost.* **3**, 1752-1762 (2005).

25 Tullemans, B. M. E., Heemskerk, J. W. M. & Kuijpers, M. J. E. Acquired platelet antagonism: off-target antiplatelet effects of malignancy treatment with tyrosine kinase inhibitors. *J. Thromb. Haemost.* **16**, 1686-1699 (2018).

26 Senis, Y. A. Protein-tyrosine phosphatases: a new frontier in platelet signal transduction. *J. Thromb. Haemost.* **11**, 1800-1813 (2013).

27 Pradhan, S., Alrehani, N., Patel, V., Khatlani, T. & Vijayan, K. V. Cross-talk between serine/threonine protein phosphatase 2A and protein tyrosine phosphatase 1B regulates Src activation and adhesion of integrin alphaIIbbeta3 to fibrinogen. *J. Biol. Chem.* **285**, 29059-29068 (2010).

28 Haining, E. J., Yang, J. & Tomlinson, M. G. Tetraspanin microdomains: fine-tuning platelet function. *Biochem. Soc. Trans.* **39**, 518-523 (2011).

29 Mangin, P. H., Kleitz, L., Boucheix, C., Gachet, C. & Lanza, F. CD9 negatively regulates integrin alphaIIbbeta3 activation and could thus prevent excessive platelet recruitment at sites of vascular injury. *J. Thromb. Haemost.* **7**, 900-902 (2009).

30 Miao, W. M., Vasile, E., Lane, W. S. & Lawler, J. CD36 associates with CD9 and integrins on human blood platelets. *Blood* **97**, 1689-1696 (2001).

31 Versteeg, H. H., Heemskerk, J. W., Levi, M. & Reitsma, P. H. New fundamentals in hemostasis. *Physiol. Rev.* **93**, 327-358 (2013).

32 de Witt, S. M. *et al.* Identification of platelet function defects by multi-parameter assessment of thrombus formation. *Nat. Commun.* **5**, 4257 (2014).

33 Ware, J., Russell, S. & Ruggeri, Z. M. Generation and rescue of a murine model of platelet dysfunction: the Bernard-Soulier syndrome. *Proc. Natl. Acad. Sci. USA* **97**, 2803-2808 (2000).

34 Bergmeier, W. *et al.* The role of platelet adhesion receptor GPIbalpha far exceeds that of its main ligand, von Willebrand factor, in arterial thrombosis. *Proc. Natl. Acad. Sci. USA* **103**, 16900-16905 (2006).

35 Verdoia, M. *et al.* Vitamin D levels and high-residual platelet reactivity in patients receiving dual antiplatelet therapy with clopidogrel or ticagrelor. *Platelets* **27**, 576-582 (2016).
